# Linking Personality Traits to Mediterranean Diet Adherence and Exploring Gene–Diet Interactions in Neuroticism

**DOI:** 10.3390/nu17233791

**Published:** 2025-12-03

**Authors:** José V. Sorlí, Carolina Ortega-Azorín, Oscar Coltell, Rebeca Fernández-Carrión, Eva M. Asensio, Olga Portolés, Alejandro Perez-Fidalgo, Judith B. Ramirez-Sabio, Javier Guillem-Saiz, José A. Costa, Ignacio M. Gimenez-Alba, Rocío Barragán, Jose M. Ordovas, Dolores Corella

**Affiliations:** 1 Department of Preventive Medicine and Public Health, School of Medicine, University of Valencia, 46010 Valencia, Spainrebeca.fernandez@uv.es (R.F.-C.); i.gimenez.alba@uv.es (I.M.G.-A.); rocio.barragan@uv.es (R.B.); 2 CIBER Fisiopatología de la Obesidad y Nutrición, Instituto de Salud Carlos III, 28029 Madrid, Spain; oscar.coltell@uji.es (O.C.); jose.ordovas@tufts.edu (J.M.O.); 3 Department of Computer Languages and Systems, Universitat Jaume I, 12071 Castellón, Spain; 4 INCLIVA Biomedical Research Institute, University Clinic Hospital of Valencia, 46010 Valencia, Spain; 5 Centro de Investigación Biomédica en Red de Cáncer (CIBERONC), Instituto de Salud Carlos III, 28029 Madrid, Spain; 6 Servicio de Oncología, Hospital de Sagunto, 46520 Sagunto, Spain; 7 Department of Psychology, International University of Valencia, 46010 Valencia, Spain; 8 Division of General Medicine, Department of Medicine, Columbia University Irving Medical Center, New York, NY 10032, USA; 9 Nutrition and Genomics Laboratory, Jean Mayer-US Department of Agriculture Human Nutrition Research Center on Aging, Tufts University, Boston, MA 02111, USA; 10 Institutos Madrileños de Estudios Avanzados (IMDEA) Nutrition Institute, Campus of International Excellence (CEI) of the Autonomous University of Madrid (UAM) + the Spanish National Research Council (CSIC), 28049 Madrid, Spain

**Keywords:** Mediterranean diet, personality traits, neuroticism, genetics, gene-diet interactions, precision nutrition

## Abstract

Background and Objectives: There is adherence to a healthy Mediterranean diet (MedDiet), but adherence varies widely. Precision nutrition is increasingly interested in individual characteristics influencing diet adherence, but few studies have examined personality traits. Our main aim was to investigate the association between personality traits and MedDiet adherence. Our secondary aims were to explore genome-wide genetic variants associated with neuroticism, including replication of previous findings, as well as to explore gene–MedDiet interactions. Methods: We analyzed participants (aged 55–75) in the PREDIMED-Plus-Valencia study and measured clinical, lifestyle, and genetic factors. The Eysenck Personality Questionnaire-Revised (EPQ-R) was used to measure neuroticism, psychoticism, and extraversion. Genotyping was undertaken, and associations with candidate SNPs, genome-wide association studies (GWAS), genetic risk scores (GRS), and gene–MedDiet interactions were explored. Results: Neuroticism was inversely (beta = −0.09; *p* = 0.001) associated with adherence to the Mediterranean diet (MEDAS-17). Likewise, the probability of low MedDiet adherence increased neuroticism (OR: 1.27; 95% CI: 1.02–1.60; *p* = 0.031 per SD). In the GWAS for this trait, several SNPs surpassed the suggestive level of statistical significance. The most strongly associated was rs10181407-*NDUFA10* (NADH dehydrogenase 1 alpha subcomplex subunit 10) (beta = −2.39; *p* = 2.70 × 10^−6^). The GRS for neuroticism was significantly associated with MedDiet adherence (beta = −0.18; *p* = 0.020), increasing the causality level. We replicated some candidate SNPs, and among them, the rs2243873-*EHMT2* (euchromatic histone lysine methyltransferase 2) gene. The analysis of gene–MedDiet interactions revealed the role of these dietary modulations. Conclusions: Neuroticism was the personality trait most inversely associated with MedDiet adherence, suggesting its integration in precision nutrition analysis. Moreover, neuroticism-related genetics and MedDiet modulations will also be important.

## 1. Introduction

Currently, in the field of precision nutrition, there is great interest in identifying individual characteristics that may influence greater or lesser adherence to dietary recommendations [1,2]. From a nutritional epidemiology perspective, the most frequently analyzed factors to assess their influence on adherence to dietary recommendations have been sex, age, education level, socioeconomic status, cultural determinants, body mass index, sleep, physical activity, alcohol consumption, tobacco use, food preferences, and the presence of diabetes or other diseases [3,4,5,6,7]. Nevertheless, there has been a limited amount of research on the role of personality traits [8,9,10]. This is because psychological factors have traditionally received less attention in nutritional research, and food patterns have rarely been evaluated concurrently from a psychological perspective. Fortunately, the scientific community is experiencing a significant increase in interest in the potential influence of psychological factors (such as personality traits) on dietary intake and disease risk [11,12]. Precision nutrition will benefit from this multi/inter-disciplinary approach, as it offers the potential to personalize and enhance dietary recommendations for prevention and treatment.

### 1.1. Personality Measurement and Assessment

While there are numerous definitions of personality, it can be considered a psychological construct that is employed to explain a variety of human behaviors through individual traits [13,14]. It takes into account individual differences in emotional, interpersonal, motivational, and other aspects [15]. Numerous personality theories and models exist in the literature [16,17]; however, the “super three” and the “big five” traits have been used more extensively in biomedical research [18,19,20,21,22,23]. According to Eysenck’s three-factor theory, there are three main components: Extraversion, neuroticism, and psychoticism [24], while the Big Five model argues that five factors are essential to explain the majority of variance in personality research: Extraversion, neuroticism, agreeableness, conscientiousness, and openness [25]. Subsequently, many studies have been conducted comparing the advantages and disadvantages of each theory, and even harmonization proposals have been put forward to integrate the results of both approaches [18,26,27,28,29]. It has been reported that neuroticism is the dimension with the most shared variance between the two approaches, as it is robustly measured in both [18,24,25,30,31,32,33]. Another aspect to consider when comparing results across studies that examine personality traits is the version of the questionnaire that was used. For both the “super three” and the “big five” traits, various versions have been designed and validated, including full-length versions as well as shorter, simplified ones to reduce the duration of administration [24,25,30,34,35,36,37,38,39,40]. However, abbreviated versions may have the limitation of measuring personality traits with less validity than the full-length versions, depending on the population.

### 1.2. Personality Traits and Nutrition

Therefore, when analyzing the associations between personality traits and dietary patterns, the type of personality questionnaire used should be considered. In general, the personality questionnaires employed in nutrition have been heterogeneous [8,9,10,11,12,41,42,43,44,45,46,47,48]. Likewise, the investigated dietary components or patterns were also varied. Among them, associations between personality traits and adherence to vegetarian diets have been reported [43,44,45]. In addition, the association of personality traits with taste and food preferences has also been analyzed, highlighting the link between neuroticism and a preference for salty taste, fatty foods, and more unhealthy dietary choices [9]. Similarly, a direct association has been found between eating plant-based foods and fish and emotional stability [10]. Another study found that neuroticism was related to a less-healthy pattern and openness with a healthier one [12]. Two interesting reviews on personality traits and dietary choices/components have provided more information on previously published works regarding demographic characteristics and findings [8,11]. Nevertheless, there is a paucity of research investigating the specific association between personality traits and adherence to the Mediterranean diet [47,48,49]. Among these studies, the Mediterranean diet pattern was assessed using three different approaches [47,48,49]. In one of them, carried out in older adults (aged 70 years) from the Lothian Birth Cohort by Mottus et al. [48], a Mediterranean-style pattern was derived from a food frequency questionnaire through PCA. In the other two studies, one carried out in adults (aged 18–65 years) attending a primary care center by Jurado et al. [47], used the 14-item Mediterranean diet adherence screener (MEDAS-14) [50] and the other [49], undertaken in adolescents (aged 14–15 years), used the Mediterranean Diet Quality Index for Children and Adolescents (KIDMED) questionnaire [51]. Likewise, personality traits were measured differently. In the Lothian Birth Cohort, the NEO Five-Factor Inventory [48] was used; whereas the Temperament and Character Inventory (TCI-125) [52] was employed in the study of adults in Spain [47]; and the Big Five Questionnaire for Children and Adolescents was administered in the study undertaken by Yañez et al. [49]. In older people, Mottus et al. [48] found that endorsing the Mediterranean-style diet dimension was associated with high openness and extraversion, and low neuroticism. Jurado et al. [47], in 206 adults, reported that adherence to the Mediterranean diet was directly associated with the character dimension of “self-directedness” and minor psychiatric morbidity. On the other hand, Yañez et al. [49], in 695 adolescents, found that higher conscientiousness was associated with a lower risk for non-adherence to the Mediterranean diet. However, extraversion was associated with a higher risk of non-adherence, and no associations with adherence were found for high levels of emotional instability.

### 1.3. Genetics of Personality Traits: Focus on Neuroticism

Furthermore, a genetic influence on personality traits has been reported [53,54,55], with associations appearing to be more prominent for neuroticism [55,56,57]. Nevertheless, after numerous genome-wide association studies (GWAS) [56,57,58,59,60,61,62,63,64,65,66,67], identifying the main genetic variants has shown considerable difficulty. Despite the numerous single-nucleotide polymorphisms (SNPs)/genes that have been reported to be significantly associated with neuroticism at the GWAS level (among them: *MAGI1*, *GRIK3*, *CRHR1*, *MAPT*, *TEM192*, *ELAVL2*, *CELF4*, *SRP9*, *PVRL3*, *PTPRE*, *BCL10*, *TRIM32*, *CADM2*, and *LINGO2*), they exhibit minimal reproducibility across investigations. The population characteristics as well as the variability of the questionnaires utilized to assess neuroticism [56,57,58,59,60,61,62,63,64,65,66,67] (mainly the abbreviated version of the EPQ, instead of the complete version) may have contributed to this.

### 1.4. Gene–Environment Interactions in Neuroticism

Furthermore, there is a growing recognition of the need to investigate not only genetics but also gene–environment interactions [68,69]. Although some gene–environment interaction studies have been conducted to investigate neuroticism, they have examined various exposures related to socio-economic status, adverse events, childhood trauma, or drug use, but diet has not been included among the environmental factors analyzed [70,71]. This provides an excellent opportunity to advance in the new field of dietary modulations of personality traits.

Therefore, our objectives were as follows: (1) Our main aim was to investigate the association between personality traits and adherence to the Mediterranean diet in this southern European population; and (2) our secondary aims were as follows: (a) To explore genetic variants, at the genome-wide level, that are associated with neuroticism in this cohort and investigate the replication of associations with previously identified candidate SNPs from a recent meta-analysis [56] in other populations; and (b) to explore gene–Mediterranean diet interactions in relation to neuroticism in this cohort, focusing on the main SNPs identified in the current study as well as in those from the meta-analysis.

## 2. Materials and Methods

### 2.1. Study Design and Participants

We conducted a cross-sectional analysis that included participants in the PREDIMED Plus-Valencia study (*n* = 465 total recruited), a field center of the multi-center PREDIMED Plus cohort, an ongoing randomized primary prevention trial in Spain [72]. The trial was registered at https://doi.org/10.1186/ISRCTN89898870. The selected participants were community-dwelling adults (men, 55–75 years; women, 60–75 years) with metabolic syndrome and a body mass index (BMI) ranging from 27 to 40 kg/m^2^ [73]. All these participants were individuals with metabolic syndrome. In this site [73], all of them were recruited from primary care centers; they were not hospitalized patients. They were individuals living at home who were not following low-calorie diets or undergoing pharmacological treatment for weight loss (exclusion criteria) [72]. In this work, adherence to the Mediterranean diet was collected at baseline. The personality questionnaire was only administered to 400 of the 465 participants, as it was not included in the study’s general protocol but was part of a specific project at the Valencia site, involving additional measures.

Written informed consent was obtained from all participants. The Human Research Ethics Committee of Valencia University, Valencia, provided approval for the study protocols and procedures in accordance with the ethical standards of the Helsinki Declaration (approval codes H1373255532771, 15 July 2013; and H1509263926814, 6 November 2017).

### 2.2. Baseline Demographic, Lifestyle, Clinical, Anthropometric, Biochemical, and Lifestyle Variables

As previously reported [72], a general questionnaire was used to evaluate socio-demographic data, lifestyle (including tobacco smoking, sleep duration on workdays, sleep duration on free days, and education), medication, and clinical characteristics at baseline. Leisure-time physical activity was assessed using the validated REGICOR short Physical Activity questionnaire [74]. The questionnaire contained questions regarding the duration, frequency (number of days), and type of activity. The total energy expenditure associated with leisure-time physical activity was estimated in Metabolic Equivalent of Tasks (METs)/min/day as a continuous variable by summing the frequency, duration, and intensity of each activity and dividing by 30 days.

The chronotype was evaluated using the Morningness–Eveningness Questionnaire (MEQ) [75]. The MEQ is regarded as an international gold standard for assessing chronotype and consists of 19 items utilizing a Likert scale. A greater total score indicated morningness. We utilized the MEQ score as a continuous variable. Type 2 diabetes was assessed as previously reported [69]. Depressive symptoms were assessed by the Beck Depression Inventory (BDI-II) [76,77], using a 21-item questionnaire with a Likert-type answer range from 0 to 3. Higher total scores in the additive scale indicate more severe depressive symptomatology.

Blood pressure was determined using a validated semiautomatic oscillometer (Omron HEM-705CP, OMRON Healthcare Europe B.V., Hoofddorp, The Netherlands) while the individual remained seated for 5 min as previously reported [72]. Calibrated scales and a wall-mounted stadiometer were employed to evaluate weight and height, respectively. Waist circumference was measured at the midpoint between the lowest rib and the iliac crest, following normal exhalation, with an anthropometric tape by trained personnel according to the PREDIMED-Plus protocol [68]. BMI was calculated by dividing weight in kilos by the square of height in meters.

Venipuncture was employed to obtain venous blood samples following an overnight fast. As previously described, fasting plasma total cholesterol, HDL-C, LDL-C, triglycerides, and glucose were assessed [73].

### 2.3. Adherence to the Mediterranean Diet

We assessed adherence to the Mediterranean diet utilizing the validated 17-item screening questionnaire from the PREDIMED-Plus study [78]. This questionnaire comprised 17 questions regarding adherence to dietary practices characteristic of the Mediterranean diet. The questions were rated as 0 (conditions not met) or 1 (criteria achieved), yielding a maximum score of 17, where higher scores reflect greater Mediterranean diet adherence. The 17-item scale is an updated version of the MEDAS-14, previously validated by our group [50]. Both scales share most items. In the PREDIMED-Plus trial, the 17-item scale (MEDAS-17) was administered to all participants, while the MEDAS-14 was only administered to those allocated to the control group at baseline. This is equivalent to fifty percent of the participants. In the present study, we focused on the MEDAS-17, but we also tested some associations with the MEDAS-14 to broaden the findings. In addition, for some specific analyses, the MEDAS-17 score was classified into 2 categorical variables: one based on the population mean (below or above 8 points) and another divided into three approximate tertiles: low adherence (≤6), medium adherence (7–9), and high adherence (10–17 points), as previously reported [73].

### 2.4. Personality Traits Assessment

The Eysenck Personality Questionnaire-Revised (EPQ-R) was used to measure the three major dimensions of personality: Neuroticism (degree of emotional stability, including anxiety, depression, and guilt), psychoticism (degree of impulsiveness, difficulty in accepting and following rules), and extraversion (degree of social engagement and openness, sociable, active, and assertive) [24]. The questionnaire also included an additional scale that measures liability (degree of social desirability). We used the full Spanish version of the EPQ-R questionnaire validated in Spain [79]. The EPQ-R questionnaire was administered in accordance with the publisher’s licensing requirements. The instrument was obtained through authorized commercial channels, and its use complied with Spanish regulations, under the supervision of a licensed psychologist. It is an 83-item self-administered questionnaire with a yes/no format derived from the complete (100-item) originally revised version in English [24]. These scales included 23 items dedicated to neuroticism, 23 to psychoticism, 19 to extraversion, and 18 to the lie dimension [76]. A score for each scale was computed by summing individual items (yes or no option depending on the dimension), resulting in a range from 0 to 23 for neuroticism and psychoticism and from 0 to 18 for extraversion. High scores indicate that individuals are more likely to have the personality trait represented by each subscale. Our psychometric data was focused on validity. We estimated the internal consistency of each dimension and, in general, we obtained valid results. We calculated Cronbach’s alpha coefficient for each of the EPQ-R domains to evaluate internal validity. The Cronbach’s alpha was 0.86 for neuroticism. This was a very high coefficient value, indicating an excellent result. Likewise, the Cronbach’s alpha was 0.75 for extraversion, showing a good coefficient. The Cronbach’s alpha for psychoticism was 0.61, suggesting a not as good validity for this dimension. These results agree with other studies carried out in Spain [39,80,81]. We did not assess the reproducibility, but, according to the study validating the EPQ-R in the Spanish population, the reproducibility test–retest, after one month, was 0.82 for neuroticism, 0.86 for extraversion, and 0.72 for psychoticism [79].

In addition, we estimated a combined factor for personality traits using factor analysis, as detailed under the Statistical Analysis subheading.

### 2.5. DNA Isolation and Genome-Wide Genotyping

Genomic DNA was isolated from the buffy coat samples using the MagNaPure LC DNA Isolation kit (Roche Diagnostics Corporation Indianapolis, IN, USA) [82]. We tested the concentration and quality of the extracted DNA using the PicoGreen equipment (Invitrogen Corporation, Carlsbad, CA, USA). Following DNA quality control, high-density genotyping was performed on samples that passed the requirements using the Infinium OmniExpress-24 v1.2 BeadChip genotyping array (Illumina Inc., San Diego, CA, USA) at the University of Valencia, Valencia, as previously reported [82,83]. Briefly, genotyping was performed in accordance with the manufacturer’s protocol and established quality standards. The Infinium OmniExpress v1.2 BeadChip encompasses approximately 710,000 markers. Allele detection and genotype assignment were carried out in the Genome Studio (Illumina, Inc., San Diego, CA, USA). Data cleaning was executed utilizing typical analysis pipelines developed in the Python programming language (ver. 3.10.10 64 bit (AMD64)), in conjunction with PLINK (ver. stable beta 7.11, 19 August) [81,82]. The overall call rate in these participants was higher than 98%. SNPs not mapped on autosomal chromosomes were filtered out. Similarly, SNPs exhibiting a minor allele frequency (MAF) below 0.01, SNPs with a low call rate (<90%), or those that diverged from the anticipated Hardy–Weinberg equilibrium (*p* < 1.0 × 10^−4^) were excluded. More than 620,000 SNPs that met the quality filter criteria were retained for subsequent investigation.

### 2.6. Statistical Analysis

For descriptive analyses, we used chi-square tests to compare proportions and Student’s *t*-tests or ANOVA to compare crude means of continuous variables. We used Pearson correlation coefficients for continuous variables and the point-biserial correlation for dichotomous variables, as well as the corresponding heatmaps, to analyze the association among selected variables.

#### 2.6.1. Associations Between Personality Traits and Adherence to Mediterranean Diet

We tested the normal distribution of 3 personality traits (neuroticism, psychoticism, and extraversion) expressed as continuous scores. We obtained the z-scores for each variable and carried out a principal component analysis (PCA) to identify a potentially lower number of latent variables, representing a combined score of the initial three traits. We calculated the Kaiser–Meyer–Olkin (KMO) value and Bartlett’s test of sphericity for the appropriateness of this analysis. We used the Kaiser criterion (components that have eigenvalues greater than 1) for selecting the optimal number of components. Finally, we obtained 3 factors, but only the first one had an eigenvalue > 1. We computed the scores for this factor, so-called “combined-factor”, and it was analyzed as a mixed variable in the association analyses. Further, we tested the association between each one of the three personality traits (expressed as original scores) as well as the combined factor with adherence to the Mediterranean diet (MEDAS-17) as a continuous variable using multivariable linear regression models sequentially adjusted for potential confounders. When indicated, models were adjusted as follows: Model 1, unadjusted; model 2, adjusted for sex, age, diabetes, and BMI; and model 3, additionally adjusted for smoking, sleep duration, physical activity, and education. Regression coefficients and p-values were computed. Further, depending on the statistical analysis, models were additionally adjusted for other variables, as detailed in the Results section. Results for MEDAS-14 were also computed for the subsample. Likewise, we analyzed the association between personality traits and low adherence (from 0 to 8 points included) to the Mediterranean diet based on the population mean. Logistic regression models were fitted to estimate the odds ratio (OR), 95% confidence intervals (CI) were computed, and ORs were expressed per SD. Models were multivariable, and we adjusted for covariates as indicated. Moreover, we depicted the association between adherence to the Mediterranean diet and neuroticism and the combined factor in multivariable adjusted models. Adjusted means and the corresponding *p*-values were estimated.

#### 2.6.2. Associations Between Genetics and Neuroticism

To identify the genes and genetic variants that are more associated with neuroticism in this population, we carried out an exploratory SNP-based GWAS. We used PLINK v1.9 and v2.0 [84,85] for the association analyses between the SNPs and neuroticism (dependent variable), fitting multivariable linear models adjusted for covariates. We considered an additive genetic effect (0, 1, or 2 copies of the variant allele). Models were sequentially adjusted for potential confounders, but we present the results for the model adjusted for sex, age, diabetes, and BMI. Additional adjustments were indicated in the text. All participants were white Caucasians, and no population stratification was detected in our previously published GWAS [83,86,87]. Here, we computed the genomic inflation factor (lambda coefficient), and no inflation was detected. Moreover, we adjusted the regression models for the 10 principal components of the 20 estimated factors computed by genomic PCA, as previously reported [83], and no effect was detected; therefore, we discarded population stratification bias, and no further adjustments for ancestry were included. We computed the corresponding *p*-values for the analyzed SNPs in the multivariable adjusted models and used the conventional thresholds of *p* < 5 × 10^−8^ for genome-wide statistical significance and *p* < 1 × 10^−5^ for the relaxing suggestive level of GWAS significance. However, since the GWAS was deemed exploratory, we also considered SNPs with *p*-values below 5 × 10^−5^ as suggestive of significance. We utilized the R QQman package to generate Manhattan plots [88]. Likewise, quantile–quantile plots (Q-Q plot) were executed in the R statistical environment to compare the expected and observed p-values as well as to estimate the lambda values [88]. We used LocusZoom-single plot to generate locus-specific graphical displays of the position of the selected SNPs in the GWAS to nearby genes and local recombination hotspots [89], as well as to estimate the linkage disequilibrium.

Also, we used FUMA (Functional Mapping and Annotation of Genome-Wide Association Studies) [90] to explore functional features at the post-GWAS level, including a gene-based GWAS analysis (which considers the aggregate effect of multiple SNPs in a single gene). FUMA SNP2GENE tools [91] were utilized for functional annotation of SNPs in genomic areas identified by lead SNPs, and the FUMA GENE2FUNC function for the annotation of genes in a biological context. We obtained the tissue expression analysis heatmap using the genes that overlap variants associated with neuroticism in the main GWAS at *p* < 1 × 10^−5^. Likewise, we used data from GTEx v8 across 54 specific tissue types in FUMA to estimate the tissue specificity for the input genes in the GWAS for neuroticism.

In addition to the analysis of individual SNPs, we selected the most significantly associated SNPs in the GWAS (considering the p-values and previous evidence of associations) to summarize the cumulative effects of genetic loci and constructed additive genetic risk scores (GRS) [92] for neuroticism. We computed an unweighted GRS by counting the number of risk alleles per SNP without considering their effect sizes. Moreover, we only considered independent SNPs (one per gene) with a relatively high allele frequency.

Furthermore, we examined whether SNPs at loci previously identified as associated with neuroticism in other populations were significantly associated with neuroticism in our participants. We selected the recent GWAS meta-analysis [56], which encompassed around 680,000 participants, representing the biggest meta-analysis of neuroticism to date. In the meta-analysis of European populations (summary statistics included in the Supplemental Table S6 of the paper published by Gupta et al. [56]), the characteristics for the 208 SNPs related to neuroticism at the genome-wide significance level (*p* < 5 × 10^−8^) were presented in that study.

#### 2.6.3. Exploratory Analysis of Gene–Mediterranean Diet Interactions on Neuroticism

Finally, we explored gene–Mediterranean diet interactions in determining neuroticism. We considered two levels of adherence to the Mediterranean diet: low (0–8 points) and high (9–17 points), as previously reported [83]. An additive genetic effect for the selected SNPs was considered, and hierarchical statistical general linear models were fitted. These models include the main variables and the corresponding interaction terms (SNP × Mediterranean diet adherence). Additionally, models were adjusted for covariates. We explored a genome-wide gene–Mediterranean diet interaction model in PLINK and specifically tested selected gene–Mediterranean diet interactions with the top significant SNPs in our GWAS, as well as with the previously reported SNPs in the published neuroticism meta-analysis [56].

#### 2.6.4. General Statistical Considerations

Our sample size was adequately powered to detect standard associations between personality traits and adherence to the Mediterranean diet. The sample size of 400 participants was predefined for logistic reasons in the PREDIMED Plus-Valencia trial context. In previous work, we estimated the statistical power for several predictors related to the Mediterranean diet and determined that a sample size of 400 individuals would be necessary to achieve 80% power for detecting an increase/decrease of 1 unit in the MEDAS-17 questionnaire (effect size) per unit of the predictor variable and a SE of 0.05 using a standard alpha of 5%. For predictors of lower SE, the effect size in the associations would be smaller. For the genetic analysis, we previously estimated [87] that, for a large genetic effect (r^2^ = 8%) on the trait, using a genetic additive model for a genetic variant with minor allele frequency (MAF) of 0.17, a sample size of at least 400 individuals was adequately powered to reach a *p*-value at the genome-wide level of *p* < 5 × 10^−8^. As the estimated genetic effect is high, we considered our GWAS analysis as exploratory, taking into account the existence of a lower effect for the genetic variants.

Statistical analyses were performed using several packages and environments, including R and IBM SPSS Statistics version 26.0, NY. All tests were two-tailed, and *p*-values < 0.05 were considered statistically significant for these associations. Correction for multiple comparisons was considered in the GWAS. The GRCh38.p14 genome build was used in the annotation of the Illumina array and GRCh37.p13 in LocusZoom.

## 3. Results

### 3.1. General Characteristics of Study Participants Including Personality Traits

Table 1 shows the general characteristics of study participants (*n* = 400) at baseline by sex, including demographic, clinical, and lifestyle factors. In general, participants were older subjects (aged 65.3 ± 4.7 years) with metabolic syndrome. Adherence to the Mediterranean diet was measured using the MEDAS-17 in all the participants, and no statistically significant differences were observed by sex. Likewise, no sex differences were observed for the MEDAS-14 score. We detected statistically significant differences by sex regarding the depressive symptomatology.

Primary education and low physical activity were higher in women, whereas current smokers were more prevalent in men (*p* < 0.05). Regarding personality traits (neuroticism, psychoticism, and extraversion), the mean score for psychoticism was low (4.55 ± 2.77 points) in comparison with the other personality traits, and no statistically significant differences per sex were detected. However, for neuroticism (10.15 ± 5.31 points), we observed statistically significant differences by sex, being higher in women than in men; *p* < 0.001. For extraversion, no statistically significant differences per sex were noted. Additionally, the use of main cardiovascular medications did not present statistically significant sex differences (global prevalence of medication use: antihypertensive drugs (78%), hypolipidemic drugs (64%), metformin (30%), and insulin (4%)).

### 3.2. Combined Factor for Personality Traits and Association Between Personality Traits and Demographic, Lifestyle, and Clinical Variables

**Figure 1:**
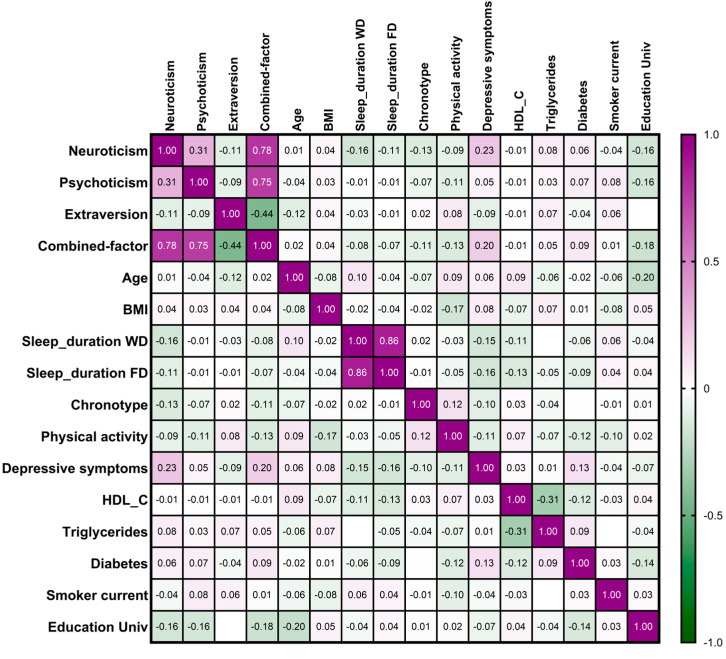
Heatmap for the correlation (Pearson) among personality traits (neuroticism, psychoticism, and extraversion), the combined factor, and demographic, clinical, and lifestyle variables. Positive or negative correlations are indicated in the color scale and by the corresponding coefficients (correlations > 0.09 are statistically significant).

First, we used PCA to analyze whether the three personality traits (scores) can be combined into higher-order factors consisting of two or more traits, thus reducing the dimensionality and creating a mixed variable. The *p*-value for Bartlett’s test was 9.6 × 10^−10^. We used the Kaiser criterion (components that have eigenvalues greater than 1) for selecting the optimal number of components. We obtained three factors, with eigenvalues of 1.36, 0.95, and 0.96, respectively. Only the first factor with an eigenvalue higher than one was selected. The percentage of variance explained by each factor was 45.4%, 31.5%, and 23.4%, respectively. We computed the scores for this so-called “combined factor” and obtained a high direct correlation with neuroticism and psychoticism, whereas the combined factor presented inverse correlations with extraversion (Figure 1). Second, we analyzed the association among the three original personality traits and the combined factor with the main demographic and clinical variables: sleep duration, morning chronotype, physical activity, smoking, and education (Figure 1).

We observed a strong direct association between neuroticism and depressive symptoms (*p* < 0.05), whereas neuroticism was inversely associated with physical activity, sleep duration, and morning chronotype. Weak associations in the same direction were observed for psychoticism, and associations in the opposite direction were detected for extraversion. No significant associations were detected for BMI, diabetes, or smoking.

### 3.3. Associations Between Personality Traits and Adherence to the Mediterranean Diet

Table 2 shows regression coefficients (beta) for the associations between personality traits (neuroticism, psychoticism, and extraversion), the combined factor, and adherence to the Mediterranean diet as continuous (MEDAS-17). Models were adjusted sequentially as indicated.

Extraversion was positively related, but not significant. Neuroticism and psychoticism were inversely associated with adherence to the Mediterranean diet in the raw model and in models adjusted for sex, age, diabetes, and BMI (model 2). However, after additional adjustment for smoking, sleep duration, physical activity, and education, the association with psychoticism did not reach statistical significance. Only neuroticism was the personality trait that remained strongly associated with less adherence to the Mediterranean diet, and this association remained even after adjustment for depressive symptoms and morning chronotype (*p* = 0.002). Additional adjustment for the main medication use did not change the statistical significance level of these associations.

Likewise, the combined factor, including a mixed score with positive correlations with neuroticism/psychoticism and inverse correlation with extraversion, remained strongly inversely associated with the Mediterranean diet in the fully adjusted model (−0.42 points per SD); *p* = 0.003. For MEDAS-14, results were similar, and neuroticism was strongly associated with low adherence to MEDAS-14 (beta: −0.077; *p* = 0.002 in model 2; and beta: −0.073, *p* = 0.005, in model 3). Likewise, the combined factor of personality traits remained statistically significantly associated with MEDAS-14, even in model 3 (beta: −0.347; *p* = 0.008). Moreover, we analyzed the risk (OR) of having a low adherence to the Mediterranean diet (MEDAS-17, less than 9 points), depending on the personality traits (Table 3).

Personality traits were analyzed as z-scores, and the ORs per SD were computed. After full multivariate adjustment, a higher neuroticism score was associated with a higher risk of low Mediterranean diet adherence (OR: 1.27; *p* = 0.031 per SD). Similarly, the combined factor was strongly associated with a higher risk of low Mediterranean diet adherence (OR: 1.36; *p* = 0.007 per SD).

### 3.4. Adherence to Mediterranean Diet and Personality Traits

Although our primary aim was to analyze the association between personality traits and the Mediterranean diet, there is also research connecting diet quality to the likelihood of displaying more altered personality traits. Therefore, we also examined the association between the Mediterranean diet and personality traits, specifically concentrating on neuroticism and the combined factor.

**Figure 2:**
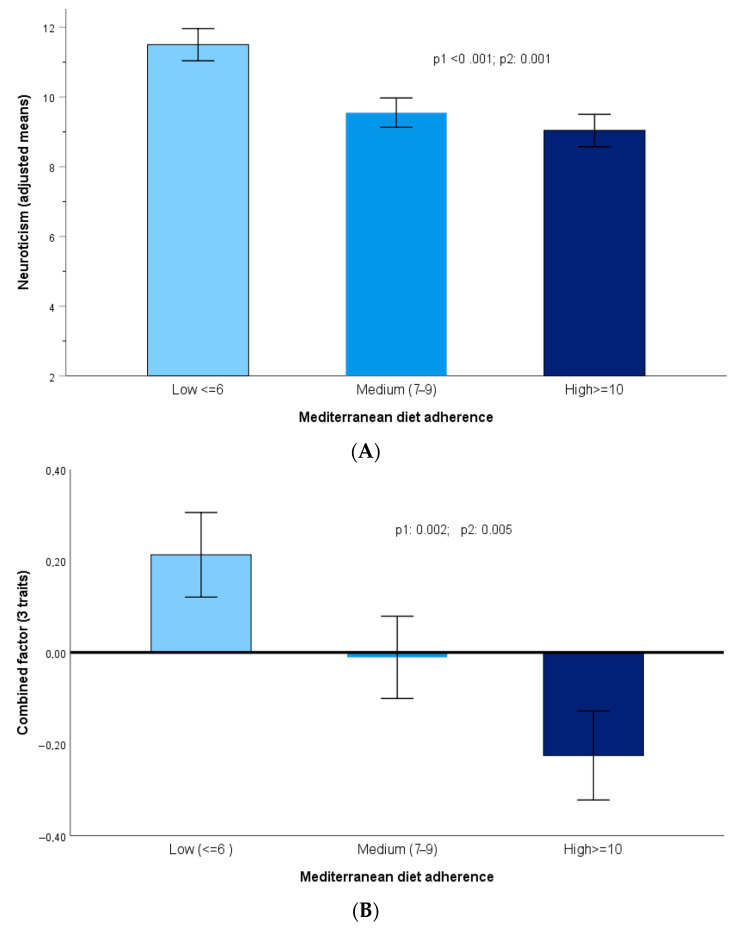
Adjusted means of neuroticism (**A**) and for the combined factor (**B**), depending on the level of adherence to the Mediterranean diet. P1: *p*-value for the association in the model adjusted for sex, age, diabetes, and BMI. P2: Model additionally adjusted for smoking, physical activity, sleep duration, and education (Error bars: SE of means).

Figure 2 shows the adjusted means of neuroticism (A) and for the combined factor (B) depending on the levels of the Mediterranean diet adherence, categorized as low (≤6), medium (7–9), and high adherence (10–17 points).

Models were multivariable adjusted as indicated in Figure 2, and the corresponding *p*-values were computed. We noted robust inverse statistically significant associations between the tertiles of the Mediterranean diet and neuroticism (*p* < 0.001) or the combined score (*p* < 0.001).

### 3.5. Genetic Factors Associated with Neuroticism: Exploratory GWAS

#### 3.5.1. Exploratory GWAS for Neuroticism

**Figure 3:**
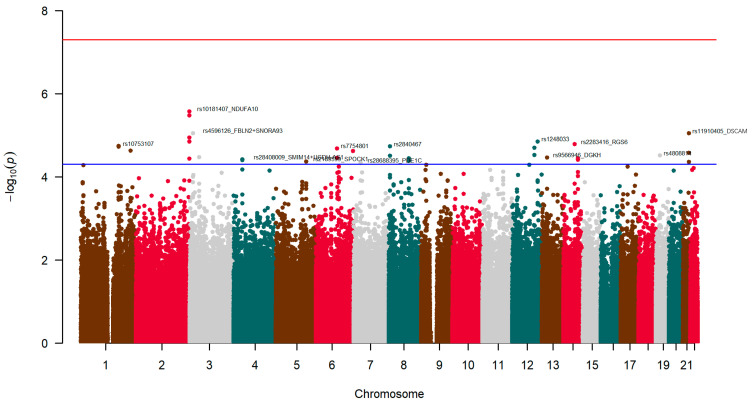
Manhattan plot for the SNP-based GWAS analysis for neuroticism adjusted for sex, age, diabetes, and BMI. The top-ranked genes with *p* < 5.0 × 10^−5^ were annotated. The red line represents the threshold 1 (−log_10_ (5 × 10^−8^)) for the GWAS statistical significance. The blue line represents the threshold 2 (−log_10_ (5 × 10^−5^)).

First, we analyzed the SNPs at the genome-wide level associated with neuroticism (as a continuous trait score). We fitted several models, and no relevant differences were detected; therefore, we presented the results for the model adjusted for sex, age, diabetes, and BMI. Figure 3 displays the corresponding Manhattan plot of the exploratory GWAS of neuroticism in this population, showing the *p*-value (−log10 *p*) of each SNP analyzed in the model adjusted for sex, age, diabetes, and BMI. Figure S1 shows the corresponding Q-Q plot. The estimated lambda (λ) value was very good (λ = 1.01), indicating no population stratification bias.

The most significantly associated SNP (at *p* 2.70 × 10^−6^) was the rs10181407, located in chromosome 2 and assigned to the *NDUFA10* (NADH dehydrogenase 1 alpha subcomplex subunit 10) gene.

**Figure 4:**
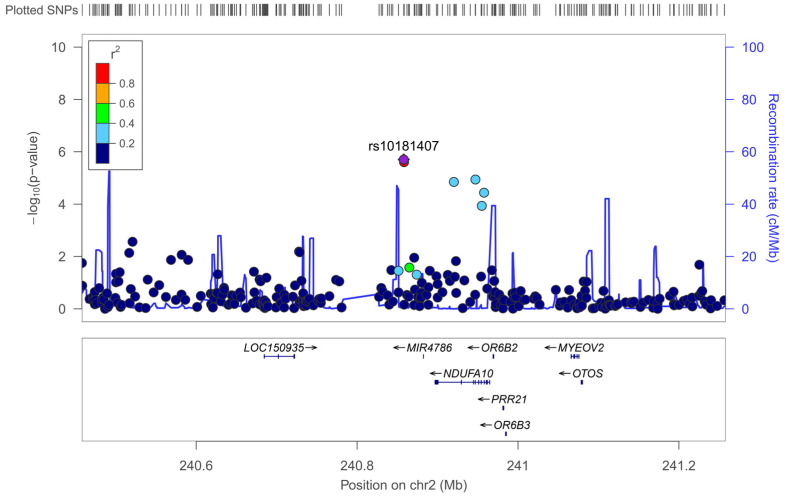
Locus zoom plot of the most significant SNP, rs10181407, in the GWAS of neuroticism in this population.

Figure 4 shows the corresponding Zoom plot for the rs10181407-*NDUFA10* SNP. Similarly, rs10933578-*NDUFA10*, in very high LD with rs10181407-*NDUFA10*, was the second most significant SNP (at *p* = 3.37 × 10^−6^). As far as we know, this is the first time that the *NDUFA10* gene has been reported to be associated with neuroticism in GWAS.

Table 4 shows more detailed information on the SNPs most significantly associated with neuroticism in the model adjusted for sex, age, diabetes, and BMI (top-ranked SNPs by *p*-value < 5 × 10^−5^).

The GWAS revealed several loci with suggestive associations at the standard 1 × 10^−5^ level, including variants in the *NDUFA10*, *FBLN2* (Fibulin-2), and *DSCAM* (Down syndrome cell adhesion molecule) genes, although none reached genome-wide significance at the 5 × 10^−8^ level.

Figure S2 shows the heatmap for gene expression corresponding to the most significant genes (DNUFA10, *FBLN2*, and *DSCAM*) based on the data set GTE × V8 (54 tissue types), showing the average expression obtained in FUMA. *NDUFA10* was highly expressed in the brain (mainly in the cerebellum and in the cerebellar hemisphere). In addition to the hits at standard *p* < 1 × 10^−5^, we also included in Table 4 the other SNPs that reached a *p*-value < 5 × 10^−5^, taking into account the exploratory characteristic of this analysis. Interestingly, we detected at this level some genes previously reported to be associated with neuroticism (or related phenotypes), including the *RGS6* (G-protein signaling 6), the *ANKS1B* (Ankyrin Repeat and Sterile Alpha Motif Domain Containing 1B), the *DGKH* (Diacylglycerol Kinase Eta), and the *PDE1C* (Phosphodiesterase 1C) genes, among other intergenic loci. The most significant intergenic SNPs were rs1248033 in chromosome 12 (see the Zoom plot in Figure S3), followed by rs10753107 in chromosome 1 (Zoom plot in Figure S4). Figure S5A shows the Manhattan plot for the gene-based GWAS analysis in FUMA, and Figure S5B displays results of the enrichment analysis of the most significant genes (GTE × V8; 54 tissue types) showing a strong enrichment of genes expressed in the different brain structures (brain putamen basal ganglia, brain caudate basal ganglia, brain frontal cortex, and brain cerebellar hemisphere, among others).

#### 3.5.2. GRS for Neuroticism

**Figure 5:**
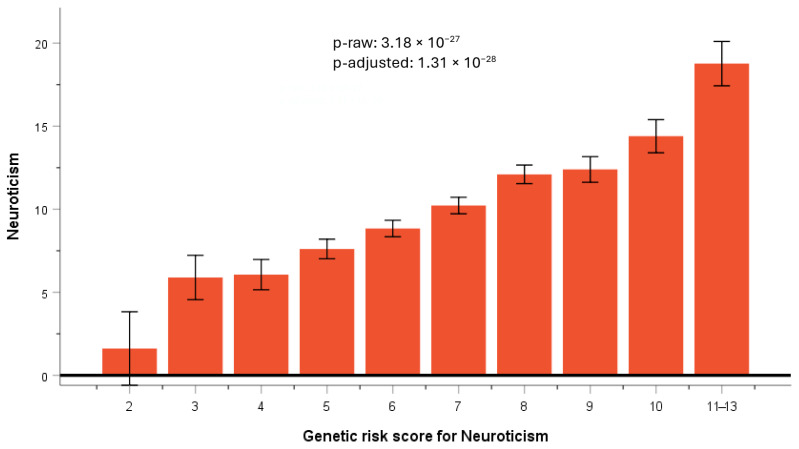
Association between the genetic risk score (GRS) for neuroticism and the neuroticism trait measured with the EPQ-R (values are means ± SE). *p*-raw: Unadjusted model. *p*-adjusted: Model adjusted for sex, age, diabetes, and BMI.

We selected the most relevant SNPs from the exploratory GWAS for neuroticism carried out in this population and constructed a GRS (unweighted). SNPs were selected taking into account the *p*-value for statistical significance (top-ranked), the independence among them, and the MAF (higher MAF preferred), as well as the previous evidence of being related to neuroticism. The selected SNPs were as follows: rs10181407-*NDUFA10*, rs4596126-*FBLN2*-*SNORA93*, rs11910405-*DSCAM*, rs1248033-intergenic, rs2283416-*RGS6*, rs2840467-intergenic, rs7958517-*ANSKS1B*, and rs9566946-*DGKH*. The score ranged from 0 to 16 points. Figure S6 shows the frequency distribution of the GRS for neuroticism in this population. The mean for the GRS score was 6.76 ± 1.88 points. As the frequency of subjects having a GRS of 12 and 13 was very low, they were included in a combined category from 11 to 13. We detected a very strong association between the GRS and neuroticism (Figure 5).

The higher the GRS, the higher the score obtained on the neuroticism scale (from 0 to 23), with *p* = 3.18 × 10^−27^ in the raw model and *p* = 1.31 × 10^−28^ in the adjusted model for sex, age, diabetes, and BMI.

Given the difficulty in determining whether the association between neuroticism and lower adherence to the Mediterranean diet in a cross-sectional study reflects a causal relationship—or, conversely, whether lower adherence to the Mediterranean diet is associated with a higher prevalence of neuroticism—having a genetic indicator associated with an increased risk of neuroticism allows us to apply Mendelian randomization [93] to better understand the relationship. Therefore, we analyzed the association between the neuroticism GRS as a proxy for neuroticism to investigate whether there is an association between this instrumental genetic variable and adherence to the Mediterranean diet. We used adherence as the dependent variable (MEDAS-17) and the genetic GRS as the predictor. In both the crude and adjusted models, we found statistically significant associations (*p* < 0.05) between the GRS and lower adherence to the Mediterranean diet (beta: −0.177; SE:0.076; *p* = 0.020 adjusted for sex, age, diabetes, and BMI), indicating a genetic association that, through the Mendelian randomization approach, may contribute to an increase in the level of causality of this association. Accordingly, after adjusting the model for the measured neuroticism variable, the association between the GRS and adherence to the Mediterranean diet did not remain statistically significant (beta: −0.059; SE: 0.089; *p* = 0.505).

#### 3.5.3. Testing for Replication of SNP Previously Reported to Be Associated with Neuroticism

We examined whether candidate SNPs previously identified as linked with neuroticism in other populations also exhibited significant associations in this population. As detailed in Methods, we selected, for replication, the recent GWAS meta-analysis [56]. We compared the 208 SNPs associated at the GWAS level in the summary statistics with our target sample (using our genotyping array) and identified 33 coincident SNPs. We examined the association between the overlapped SNPs and neuroticism in a model adjusted for sex, age, diabetes, and BMI.

Table 5 displays the top five SNPs most associated in our population, ranked by *p*-values. Only three SNPs reached the statistical significance at *p* < 0.05 needed for replication. A complete list including the 33 variants can be found in Table S1.

Interestingly, two of the three SNPs were discovered for the first time in the meta-analysis, and this is the first time that the association with neuroticism in a different population has been reported. The first one was rs12407512-intergenic in chromosome 1, and the second one was rs2243873 in the *EHMT2* (euchromatic histone lysine methyltransferase 2) gene. For the three SNPs, we replicated the beta direction, although the effect was higher in our population.

In our study, we always reported the beta for the minor allele to better compare the results. However, in the meta-analysis, the beta was reported for the “effect allele” (sometimes the minor and other times the major). Therefore, for the rs2243873-*EHMT2* SNPs, the authors [56] reported a positive beta for the effect allele (major), and we reported a negative beta for the minor allele. Thus, the minor allele in both populations was inversely associated with the neuroticism score.

#### 3.5.4. Gene x Mediterranean Diet Interactions in Determining Neuroticism

**Figure 6:**
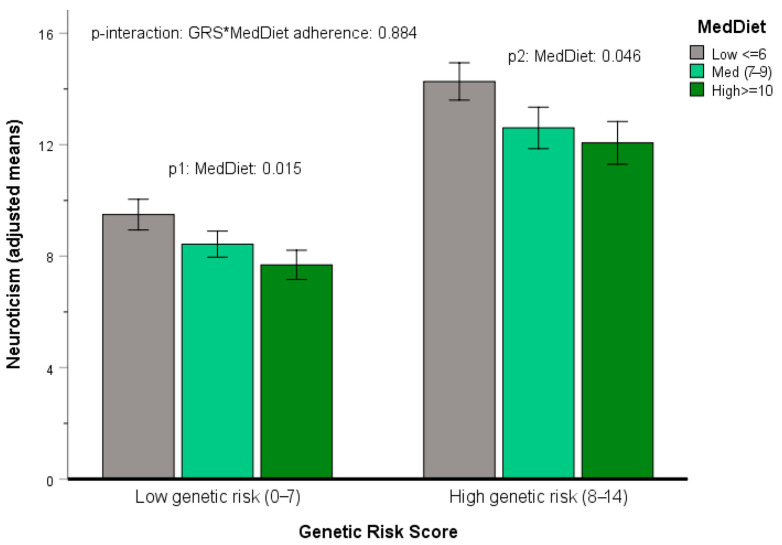
Gene–Mediterranean diet (MedDiet) interaction between the GRS (2 levels based on the mean: low [0–7] and high [8–16]) and the adherence to MedDiet (3 levels). *p*-interaction: *p*-value for the interaction term GRS*MedDiet adherence in the multivariable model adjusted for sex, age, diabetes, and BMI. p_1_: *p*-value for the MedDiet in the strata of the low genetic risk-adjusted model. p_2_: *p*-value for MedDiet in the genetic strata of the high genetic risk adjusted model.

Finally, we explored gene–Mediterranean diet interactions for determining neuroticism. Considering the limitations of our sample size, we focused our aim on selected analysis. Initially, we analyzed the most significant SNPs linked to neuroticism in our GWAS, aggregated into the calculated GRS to minimize the number of comparisons. Figure 6 illustrates the adjusted means of neuroticism depending on the genetic risk score (GRS), categorized into two levels, low genetic risk and high genetic risk, based on the mean score, along with adherence to the Mediterranean diet, which is categorized into three categories, as previously detailed.

The interaction between the SNP and adherence to the Mediterranean diet was evaluated in a multivariable linear regression model, incorporating main effects, interaction terms, and adjustments for other factors. Although a statistically significant interaction term was not detected (*p* = 0.884), adherence to the Mediterranean diet was associated with reduced neuroticism levels in both the low genetic risk group (*p* = 0.015) and in the high genetic risk group (*p* = 0.046), indicating the lack of genetic determinism (a so-called biological interaction [94]). To better understand this interaction, we focused on one SNP.

**Figure 7:**
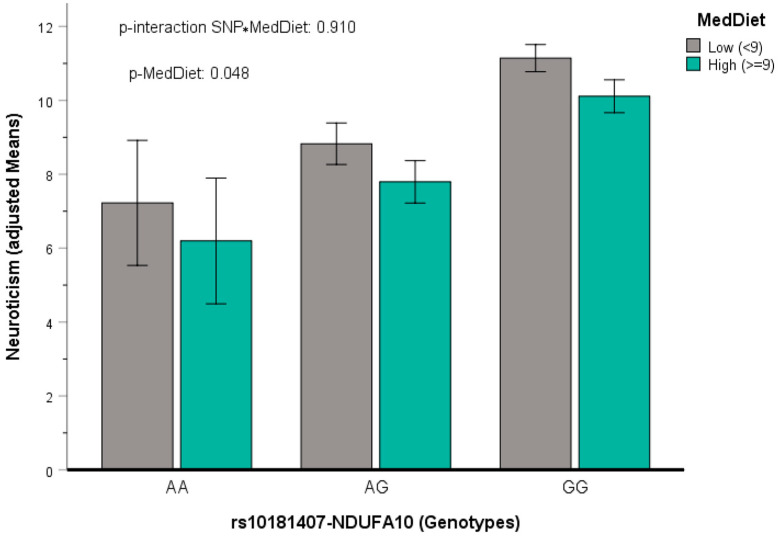
Gene–Mediterranean diet (MedDiet) interaction between the SNP rs10181407-*NDUFA10* and the adherence to MedDiet (2 levels). *p*-interaction: *p*-value for the interaction term SNP*MedDiet in the hierarchical model adjusted for sex, age, diabetes, and BMI. *p*-MedDiet: *p*-value for the MedDiet term in the adjusted model.

We selected the rs10181407-*NDUFA10*, and adherence to the Mediterranean diet was tested as dichotomous (Figure 7).

For this SNP, we analyzed the three genotypes in a multivariable gene–diet interaction model adjusted for covariates. Accordingly, the interaction term was not statistically significant (*p* = 0.910), but the Mediterranean diet reached statistical significance (*p* = 0.048), showing the lack of genetic determinism.

Moreover, we explored, at the genome-wide level, the existence of statistically significant gene–Mediterranean diet interactions as described in Methods. Adherence to the Mediterranean diet was considered at two levels: Figure S7 shows the Manhattan plot for the GWAS analyzing the *p*-values of the interaction terms. Both the GWAS threshold for statistical significance and the suggestive threshold were depicted. None reached the GWAS level of significance, but some surpassed the suggestive level of statistical significance. Among them, the hit SNP was rs2152134-intergenic in chromosome 1 (*p*-value-interaction *p* = 3.5 × 10^−7^). This SNP is located near the *CTH* and the *PTGER3* genes. The minor allele was associated with a small increase in the neuroticism score when adherence to the Mediterranean diet was low. However, when adherence was high, the minor allele was related to a decrease in neuroticism. Nevertheless, this is an exploratory analysis, and additional work will be needed to better characterize this and the other suggestive gene–diet interaction.

Finally, we focused on the analysis of gene–Mediterranean diet interactions, looking at the 33 SNPs extracted from the GWAS meta-analysis using the same hierarchical interaction model. Table 6 shows the information on the top five SNPs that had the most significant gene–diet interactions.

We detected three SNPs that presented statistically significant gene–Mediterranean diet interactions. The first one was intergenic, followed by rs3741475 in the *NOS1* (Nitric oxide synthase 1) gene and rs2155281 in the *NCAM1* (Neural cell adhesion molecule 1) gene. Table S2 provides more information about the other 33 SNPs tested.

## 4. Discussion

In this work, we have carried out a multidisciplinary approach in precision nutrition, integrating the analysis of personality traits with adherence to the Mediterranean diet and the investigation of genetic factors, also exploring gene–diet interactions. No prior research has been identified that integrated these factors. Some studies focused on the link between personality traits and diet [8,9,10,11,12,41,42,43,44,45,46,47,48], while others examined the genetic factors associated with personality traits [53,54,55,56,57,58,59,60,61,62,63,64,65,66]. However, genetics have not been included in the dietary studies mentioned, nor has diet been considered in the genetic analyses of personality. This gap presents a unique opportunity for future research to explore how these variables interact. By considering both genetic influences and dietary patterns, researchers could uncover new insights into the complex relationship between personality and health outcomes in the new era of precision nutrition. Regarding the specific association between personality and diet, the present work investigating personality traits in an elderly population in Southern Europe is also novel and of relevance, as we have detected a robust inverse association between neuroticism and adherence to the Mediterranean diet.

Although several studies have analyzed the intake of specific foods, food groups, macronutrients, taste preferences, or dietary habits [8,9,10,41,42,43,44,45,46], hardly any study has specifically examined the Mediterranean diet pattern [47,48,49]. Moreover, a recent meta-analysis [11] has emphasized the need for further research on the effect of personality on adherence to the Mediterranean diet, as there is a limited number of published articles. The three previously published investigations [47,48,49] analyzing this relationship reported mixed results. In the study carried out in older participants [48], an inverse association between neuroticism and the Mediterranean diet was reported. However, this association was not detected in adolescents [49], and other personality components were investigated in the study undertaken in primary care participants [49]. In light of these prior findings, we can state that our investigation is the first to demonstrate a clear and robust association between the neuroticism score and lowered adherence to the Mediterranean diet, using both the MEDAS-17 [74] and the MEDAS-14 [50] questionnaires. In addition to neuroticism, we also investigated the association of psychoticism and extraversion as separate traits, as well as combined in a latent variable computed by PCA. This combined score presented a direct correlation with neuroticism and psychoticism and an inverse correlation with extraversion. Using this combined factor as a personality trait variable, we obtained an inverse association with adherence to the Mediterranean diet that remained statistically significant even after multivariable adjustment. More studies are needed to compare the effect of the combined factors for personality in other populations.

Although we have already mentioned that there are very few studies that have specifically investigated the association between personality traits and Mediterranean diet adherence, it is interesting to note that some studies have reported associations between neuroticism and other personality traits with various dietary components, including fruit, vegetable, and fish intake; sweets; red meat; or sugar-sweetened beverages [8,9,10,12,41,42,43,44,45,46,48,95,96,97,98]; all of these listed items are in line with the characteristics of the Mediterranean pattern [99]. However, at the meta-analysis level, the evidence is still scarce. Thus, a recent meta-analysis [11] has reported that neuroticism had a statistically significant negative association with fruit and vegetable intake, whereas extraversion presented a positive association with fruit and vegetables. However, no significant meta-analysis effects were observed for neuroticism or extraversion associated with meat intake, high-sugar food intake, fast food, potato chips, or dairy products, highlighting as an important limitation the high heterogeneity and the small number of studies [11]. Our research is contributing by generating additional data that will allow us to better understand these associations. Although more standardization in the field is needed to better characterize the associations, the results obtained may be beneficial for interventions that aim to increase adherence to the Mediterranean diet pattern, as individuals with different personality scores may respond more or less favorably to diet advice. Further research is needed to broaden the findings of other populations with different characteristics including sex, age, socio-economic status, region, etc., and to investigate the impact of this personality trait on personalized dietary recommendations in order to enhance adherence to this healthy diet.

Neuroticism has long been considered one of the most essential domains of personality and is now becoming central to psychopathology [100]. It affects many emotional and physical health issues, making it a major public health issue [100,101,102,103]. It is characterized by a greater propensity to experience negative emotions, as well as emotional instability, anxiety, and irritability [100,104] that can influence health behaviors and dietary choices [9,105]. Individuals scoring high in neuroticism often exhibit lower emotional regulation that may impair their ability to maintain consistent adherence to a structured dietary pattern [11], such as the Mediterranean diet. Consequently, our results contribute to the idea that neuroticism may act as a psychological barrier to long-term dietary adherence that should be considered within the framework of precision nutrition.

In light of the potential significance of neuroticism in the emerging field of precision nutrition [106], it is essential to have an extensive understanding of the genetic factors and their potential modulation by environmental factors. Neuroticism has been the subject of extensive genetic research [49,67,107,108,109,110,111,112,113,114]. The heritability of neuroticism has been estimated in the range from 30% to 50% based on twin studies [111,112]. However, further GWAS were able to identify a very small % of genetic heritability based on the discovered SNPs [55,56,57,58,59,60,61,62,63]. Initially, neuroticism genetics concentrated on functional candidate genes, and subsequently, on GWAS and exome sequencing, incorporating ever-larger numbers of participants and expanding the number of SNPs identified, leading to significant advancements [53,54,55,56,57,58,59,60,61,62,63,64,65,66,67,107,108,109,110,111,112,113,114]. However, following an extensive review of results from many GWAS that identified over 500 SNPs linked to neuroticism, and in agreement with some researchers [55,108,111], we concluded that the genetics of neuroticism remains in its early phases, requiring further investigation. Currently, there seems to be a lack of consistency regarding relevant SNPs across many populations. In addition to the different characteristics of the populations analyzed, a relevant contributing factor, potentially related to poor consistency in the results, has been the heterogeneity in the definition of the neuroticism phenotype as stated by other authors [108,111,115]. For example, in “The Genetics of Personality Consortium”, the heterogeneity of the questionnaires was harmonized across all 29 discovery cohorts [58]. However, the large GWAS, carried out in more than 63,500 participants from 29 discovery cohorts and in more than 9700 participants from the replication cohort, was only able to discover one genome-wide significant SNP in the *MAGI1* gene (rs35855737), and it was not replicated. Considering the phenotype limitations, other studies have combined cohorts using the same questionnaire to define neuroticism. The UK Biobank conducted the largest single GWAS sample for neuroticism and the most homogeneous in terms of the assessment of the neuroticism phenotype. They used the 12 items of the neuroticism scale from the EPQ-R short form [58] and selected another three cohorts using the same instrument for neuroticism in undertaking a combined GWAS. However, no good replication results were observed in the separate analyses.

One advantage of our study is that we have used the complete EPQ-R questionnaire, and we have analyzed 23 items for neuroticism (instead of the 12 of the UK Biobank), potentially increasing the validity of the assessed phenotype. Thus, we have conducted an exploratory GWAS for neuroticism in this Mediterranean population, since no prior studies are available for it. Our sample is low, but it can at least serve to detect associations (if any) with large, clinically relevant effects. In previous GWAS in our cohort, we have been able to find statistically significant associations at the GWAS level for serum bilirubin (UGTs genes) [87], for bitter taste (*TAS2R38* gene) [82], and for serum polyunsaturated fatty acids (FADS genes) [80], among others [86]. However, for neuroticism, although we obtained interesting results, we did not detect any SNPs associated at the standard GWAS level of significance (*p* < 5 × 10^−8^). We did detect several SNPs at the standard suggestive level of GWAS significance (*p* < 1 × 10^−5^). The SNP most significantly associated with neuroticism in this population was the rs10181407-*NDUFA10* at *p* = 2.70 × 10^−6^. As far as we know, this is the first time that this SNP and the *NDUFA10* gene have been associated with neuroticism. Nevertheless, it is a good candidate. It encodes a subunit of the mitochondrial complex I with activity NADH dehydrogenase and oxidoreductase, and is an emerging metabolism-related gene. Abnormal expression of *NDUFA10* contributes to the assembly disorder of mitochondrial complex I and affects the electron respiratory chain, contributing to several neurodegenerative diseases [116,117,118,119,120,121]. More research is needed to better characterize the *NDUFA10* gene with neuroticism. The other top-ranked SNP at the suggestive standard GWAS level of significance was located in the *DSCAM* gene. This gene, identified from the “Down syndrome critical region” on chromosome 21, plays a significant role in neural circuit formation during development as a cell adhesion molecule with extensively documented multifaceted functionalities [122]. Recently, downregulation of this neurodevelopmental gene was associated with disruptions in synaptic plasticity and was identified as a key gene in pathways and molecular targets associated with post-traumatic stress disorder [123]. Also, altered function in the *DSCAM* gene has been related to autism spectrum disorders [124] and other neurological alterations [125,126]. The *FBLN2* gene attained statistical significance at the suggestive GWAS threshold. This gene encodes a secreted extracellular matrix (ECM) glycoprotein associated with various cancers and other neural processes [127,128,129]. It may contribute to organ development, particularly during the differentiation of neural structures [129].

Additionally, among the top significantly associated genes at the exploratory GWAS level (*p* < 5 × 10^−5^), we have identified some genes previously reported to be associated with neuroticism. This includes the RGS6 gene, which was previously identified as 1 of 23 loci with pleiotropic effects on four or more human psychiatric disorders and associated with intellectual disability and other phenotypes related to neurodegeneration [130,131,132]. Likewise, for the *ANKS1B,* previous GWAS reported that this gene is associated with neuroticism in the UK Biobank after a gene-based GWAS analysis [66]. Similarly, we have obtained as top-ranked in this population the *DGKH* gene, which is a well-known gene for bipolar disorders but is also associated with personality traits in some studies [55,133,134,135]. Despite the constraints of our exploratory GWAS, the quality control implemented at each analytical stage was adequate, generating consistent results for the discovered SNPs. Moreover, functional analysis revealed genes exhibiting enhanced expression in brain tissues, an observed characteristic among numerous genes linked to neuroticism SNPs.

Therefore, based on these results for the SNPs identified as most significant, we constructed a population-specific GRS. Although summary statistics for the most significant SNPs associated with neuroticism in several cohorts [55,56,57,62,64] have been published, the utility of these GRS is still very low. Thus, it has been reported that GRS derived from the primary UK Biobank sample captured less than 1% of the variance in neuroticism in the GS:SFHS and QIMR cohorts tested in the same study [56].

While our primary objective was to examine the relationship between personality traits and adherence to the Mediterranean diet, it is also recognized that the Mediterranean diet plays a role in the increased or decreased risk of many diseases [136,137,138,139]. In this case, it is also of interest to investigate whether a greater or lesser adherence to the Mediterranean diet is associated with neuroticism. By examining this relationship, we have observed a statistically significant association between a higher adherence to the Mediterranean diet and a lower Neuroticism score. However, as a cross-sectional study, it is not possible to determine whether neuroticism is associated with a lower adherence to the Mediterranean diet, or whether the lower adherence to the Mediterranean diet increases the risk of neuroticism, or both. One possibility to increase the level of causality or better understand the direction of the effect is to conduct Mendelian randomization approaches [90,140,141]. These use genetic variants as a proxy for exposure and test the causal effect of the variants (instrumental variable) on an outcome. In this case, we have identified a set of genetic variants highly associated with neuroticism as a good instrument of this variable in the association with adherence to the Mediterranean diet (outcome), instead of using previously published GRS for neuroticism obtained in other populations. Numerous studies have emphasized the biases and inadequacies of GRS in predicting outcomes for individuals from populations distinct from those utilized in their creation [142,143,144]. Despite being a European population, our Mediterranean cohort exhibits some distinct genetic characteristics compared to northern and central Europe [145]. Furthermore, environmental factors may vary among these populations and influence the genetic effects. Thus, we constructed an exploratory GRS using the most statistically significant SNP as well as other SNPs in top-ranked genes previously linked with neuroticism. In total, eight SNPs were included. As expected, we observed a strong association between this GRS and neuroticism in our population, as well as a statistically significant association between exploratory GRS and adherence to the Mediterranean diet. This genetic association can contribute to increasing the level of causality of the cross-sectional association between measured neuroticism and adherence to the Mediterranean diet. Moreover, this neuroticism GRS can be used by other researchers in this country to test the genetic associations in order to replicate our results.

Further, we tested the potential replication in our Mediterranean population of the association with neuroticism with the 33 SNPs previously reported as associated with this trait in the large GWAS meta-analysis [56]. Of the 33 SNPs, we were only able to replicate 3, but the important finding is that the direction of the effect was the same. Of the three replicated SNPs, two were intergenic and one was located in the *EHMT2* gene. It is important to highlight this replication because the association of this SNP with neuroticism was reported for the first time in the meta-analysis and is, therefore, being replicated for the first time in another population. The *EHMT2* gene is a very interesting candidate involved in methylation [146,147]. Further studies in other populations and functional characterization are also required.

Finally, also as exploratory, we analyzed selected gene–diet interactions. Currently, there is great interest in examining the effects of the environment on the genetic risk of neuroticism. However, the published studies examined various environmental exposures related to socioeconomic status, adverse events, childhood trauma, drug use, etc., [69,70], but the influence of diet has not been analyzed. Therefore, in the present study, we obtained the first preliminary results of some gene–diet interactions. The main limitation is that our sample size is very small, and it is necessary to replicate the results in larger cohorts. Nevertheless, as preliminary results, we have detected interesting findings. For the top-ranked SNPs in our GWAS, we have been able to observe that there is no genetic determinism for the analyzed SNPs. The association effect for each genotype depends on the level of the Mediterranean diet adherence and not only on the genetic variant. Whether in GRS analysis or even with individual SNPs, a higher genetic risk of neuroticism is not always associated with a higher neuroticism score. Greater adherence to the Mediterranean diet counteracted genetic risk. This modification of the genetic effect, but without statistical significance for the interaction term, has been called biological interaction, since there is an environmental factor (in this case diet) that can alter genetic susceptibility. More details on this type of interaction can be found in our publication on nutritional genomics [94]. On the other hand, at the genome-wide level, we have detected other gene–Mediterranean diet interactions with suggestive statistically significant interaction terms that need further investigation. Specifically, it would be interesting to examine the three statistically significant gene–Mediterranean diet interactions (the top-ranked) observed when analyzing the 33 candidate SNPs included in the replication list. However, we will not dwell on them further, as this is a preliminary exploratory study that needs to be expanded to other populations.

## 5. Conclusions

After studying the association between personality traits and adherence to the Mediterranean diet, we can conclude that neuroticism is the most significantly associated trait in this older Mediterranean population, exhibiting an inverse relationship and supporting previous findings. Additionally, we have analyzed a combined factor of the three personality traits, which exhibit interesting associations that warrant further investigation. The magnitude of the association between neuroticism and adherence to the Mediterranean diet is important, and we suggest that this trait be included in more studies in the field of precision nutrition, as it is an understudied characteristic in most of the research conducted. Furthermore, we have identified several genetic variants strongly associated with neuroticism in this population that differ from those previously reported. We have also identified some replications of genetic associations and have constructed a GRS for neuroticism that is associated with adherence to the Mediterranean diet. Therefore, new studies with multidisciplinary teams are needed to extend these findings and better understand the relationship between personality traits, adherence to the Mediterranean diet, and their genetic and environmental modulations, thereby advancing the emerging field of precision nutrition.

## Figures and Tables

**Table 1:** Demographic, clinical, and lifestyle characteristics of the study population according to sex.

	Total	Men	Women	*p* ^1^
	(*n* = 400)	(*n* = 169)	(*n* = 231)	
Age (years)	65.33 (4.71)	64.27 (5.22)	66.10 (4.14)	<0.001
Body mass index (Kg/m^2^)	32.14 (3.51)	32.04 (3.33)	32.21 (3.63)	0.629
Waist (cm)	105.50 (9.90)	110.81 (8.69)	101.60 (8.88)	<0.001
Systolic blood pressure (mmHg)	141.50 (17.79)	144.26 (18.21)	139.49 (17.23)	0.008
Diastolic blood pressure (mmHg)	80.78 (9.90)	82.54 (10.40)	79.49 (9.33)	0.002
Fasting glucose (mg/dL)	112.78 (26.59)	114.72 (30.08)	111.35 (23.68)	0.211
Total cholesterol (mg/dL)	196.52 (38.02)	187.40 (38.52)	203.18 (36.31)	<0.001
Triglycerides (mg/dL)	142.31 (62.45)	139.18 (56.35)	144.60 (66.59)	0.392
Sleep duration WD	6.75 (1.10)	6.89 (1.07)	6.64 (1.11)	0.026
Sleep duration FD	7.10 (1.15)	7.25 (1.11)	6.99 (1.18)	0.027
Physical activity (MET.min/wk)	1697 (1546)	1984 (1830)	1487 (1263)	0.001
MEDAS-17	8.01 (2.78)	7.89 (2.87)	8.10 (2.71)	0.461
MEDAS-14	8.14 (1.79)	8.34 (2.00)	7.98 (1.60)	0.167
Morning chronotype	55.99 (7.85)	57.17 (7.68)	55.12 (7.88)	0.011
Depressive symptoms	8.92 (6.19)	6.95 (5.20)	10.35 (6.46)	<0.001
Neuroticism	10.15 (5.31)	8.68 (4.98)	11.22 (5.31)	<0.001
Psychoticism	4.55 (2.72)	4.25 (2.62)	4.77 (2.77)	0.057
Extraversion	12.22 (3.62)	12.58 (3.51)	11.95 (3.68)	0.084
Current smoker (%)	10.3	15.4	6.5	<0.001
Diabetes (%)	38.8	39.1	38.5	0.915
Primary education (%)	62.8	49.1	72.7	<0.001
University education (%)	17.0	21.3	13.9	<0.001

Values are mean (SD) for continuous variables. ^1^: *p*-value for the comparisons (means) between men and women. WD: Working days. FD: Free days. MEDAS-17: Adherence to the Mediterranean diet using a 17-item score. MEDAS-14: Adherence to the Mediterranean diet using a 14-item score in a subsample (n = 192).

**Table 2:** Association between personality traits and adherence to the Mediterranean diet score.

Personality Trait	Beta ^1^ (SE)	*p* ^1^	Beta ^2^ (SE)	*p* ^2^	Beta ^3^ (SE)	*p* ^3^
Neuroticism	−0.090 (0.026)	0.001	−0.097 (0.027)	<0.001	−0.089 (0.027)	0.001
Psychoticism	−0.106 (0.051)	0.038	−0.103 (0.051)	0.047	−0.065 (0.051)	0.222
Extraversion	0.033 (0.038)	0.391	0.040 (0.039)	0.300	0.044 (0.038)	0.247
Combined factor	−0.498 (0.138)	<0.001	−0.529 (0.142)	<0.001	−0.429 (0.143)	0.003

Regression coefficients (beta) adjusted for the covariates. ^1^: Unadjusted model. ^2^: Model adjusted for sex, age, diabetes, and BMI. ^3^: Model additionally adjusted for smoking, physical activity, sleep duration, and education.

**Table 3:** Personality traits and risk of low adherence to the Mediterranean diet.

Personality Trait	OR ^1^ (95% CI)	*p* ^1^	OR ^2^ (95% CI)	*p* ^2^
Neuroticism	1.30 (1.05–1.60)	0.015	1.27 (1.02–1.60)	0.031
Psychoticism	1.33 (1.07–1.64)	0.009	1.23 (0.99–1.54)	0.064
Extraversion	0.86 (0.70–1.06)	0.166	0.86 (0.70–1.07)	0.175
Combined factor	1.44 (1.16–1.79)	0.001	1.36 (1.09–1.70)	0.007

Odds ratios (ORs) adjusted for the covariates indicated in each model. ^1^: Model adjusted for sex, age, diabetes, and BMI. ^2^: Model additionally adjusted for smoking, physical activity, sleep duration, and education. Personality traits expressed as z-scores OR: risk of low adherence (MEDAS-17 < 9) per SD of the trait.

**Table 4:** Top SNPs ranked by statistical significance in the GWAS of neuroticism adjusted for sex, age, diabetes, and BMI.

SNP	CHR	BP	A1	Beta	*p*	Gene Symbol	MAF 1	MAF 2
rs10181407	2	240857988	A	−2.392	2.70 × 10^−6^	*NDUFA10*	0.154	0.238
rs10933578	2	240858334	A	−2.368	3.37 × 10^−6^	*NDUFA10*	0.155	0.238
rs4596126	3	13659897	C	2.087	9.00 × 10^−6^	*FBLN2, SNORA93*	0.202	0.394
rs11910405	21	42077795	C	−1.97	9.03 × 10^−6^	*DSCAM*	0.188	0.216
rs3792089	2	240947066	A	−2.338	1.14 × 10^−5^	*NDUFA10*	0.150	0.072
rs1248033	12	114881294	A	−1.642	1.42 × 10^−5^	intergenic	0.397	0.120
rs967476	2	240920291	A	−2.300	1.43 × 10^−5^	*NDUFA10*	0.153	0.101
rs2283416	14	72607322	G	1.837	1.66 × 10^−5^	*RGS6*	0.243	0.298
rs10753107	1	171398925	G	2.076	1.83 × 10^−5^	intergenic	0.178	0.139
rs2840467	8	5798398	G	2.899	1.87 × 10^−5^	intergenic	0.082	0.135
rs6682065	1	171397553	A	2.075	1.91 × 10^−5^	intergenic	0.182	0.139
rs7958517	12	99837070	G	1.745	2.03 × 10^−5^	*ANKS1B*	0.327	0.459
rs7754801	6	96301487	A	−1.683	2.09 × 10^−5^	intergenic	0.301	0.163
rs6426154	1	224909092	G	1.660	2.35 × 10^−5^	*CNIH3*	0.351	0.466
rs13200002	6	168577460	A	2.149	2.40 × 10^−5^	intergenic	0.147	0.198
rs2183578	21	42078727	A	−1.705	2.68 × 10^−5^	*DSCAM*	0.265	0.274
rs7303478	12	99827565	C	1.721	3.00 × 10^−5^	*ANKS1B*	0.321	0.440
rs4808814	19	18637610	A	1.604	3.09 × 10^−5^	intergenic	0.325	0.452
rs2703312	8	5818128	A	2.805	3.15 × 10^−5^	intergenic	0.083	0.195
rs33510	3	42391480	G	1.814	3.41 × 10^−5^	intergenic	0.244	0.245
rs9566946	13	42820124	A	1.689	3.46 × 10^−5^	*DGKH*	0.282	0.293
rs2799665	6	96309043	A	−1.616	3.52 × 10^−5^	intergenic	0.326	0.440
rs1483373	8	90186443	A	−3.750	3.60 × 10^−5^	intergenic	0.049	0.261
rs9323788	14	86850171	G	1.612	3.63 × 10^−5^	intergenic	0.296	0.281
rs10804402	2	240957801	A	−2.171	3.66 × 10^−5^	*NDUFA10*	0.159	0.161
rs28408009	4	39566499	G	1.835	3.85 × 10^−5^	*SMIM14*	0.214	0.411
rs17122386	14	86916425	G	1.621	3.92 × 10^−5^	*Intergenic*	0.285	0.238
rs10019815	4	39559690	A	1.851	4.02 × 10^−5^	*SMIM14*	0.216	0.389
rs7842444	8	90193247	G	−3.88	4.16 × 10^−5^	intergenic	0.045	0.264
rs2189599	5	136409585	A	1.929	4.33 × 10^−5^	*SPOCK1*	0.192	0.370
rs6517607	21	42067941	G	−1.799	4.44 × 10^−5^	*DSCAM*	0.199	0.224
rs28688395	7	31812761	G	1.772	4.64 × 10^−5^	*PDE1C*	0.251	0.416

CHR: Chromosome. BP: Base-pair. A1: Allele 1 (minor). Beta: Regression coefficient. *p*: *p*-value in the model adjusted for sex, age, diabetes, and BMI. MAF: Minor allele frequency: (1) calculated in this population, and (2) reported for CEU, CHB, JPT, and YRI populations.

**Table 5:** Replication analysis of the association between candidate SNPs for neuroticism [52] in this population. Top 5 SNPs (ranked by the *p*-value of the association) in the model adjusted for sex, age, diabetes, and BMI, compared with the meta-analysis of other populations.

SNP	CHR	BP	A1	Valencia MAF	Valencia Beta	*p* ^1^	Genes	Meta EAF	Meta Beta	*p* ^2^
rs12407512	1	217344002	C	0.132	−1.122	3.67 × 10^−2^	intergenic	0.840	0.016	3.12 × 10^−8^
rs2243873	6	31863433	C	0.352	−0.794	4.49 × 10^−2^	*EHMT2*	0.575	0.012	3.29 × 10^−8^
rs4585149	3	157493952	A	0.209	−0.864	4.83 × 10^−2^	intergenic	0.177	−0.017	1.00 × 10^−8^
rs1187257	18	35288227	G	0.243	0.805	6.03 × 10^−2^	intergenic	0.715	−0.014	2.04 × 10^−8^
rs7025144	9	120496387	A	0.255	0.792	6.29 × 10^−2^	intergenic	0.272	0.018	5.20 × 10^−13^

CHR: Chromosome. BP: Base-pair. A1: Allele 1 (minor). EAF: Effect allele frequency. Beta: Regression coefficient. Valencia MAF: Minor allele frequency calculated in this population. Meta EAF: Frequency of the effect allele (EA) in the meta-analysis [52]. ^1^ *p*-value of the model adjusted for sex, age, diabetes, and BMI. ^2^ *p*-value in the meta-analysis of other populations [52].

**Table 6:** Top 5 SNPs ranked by the p-value of the gene–Mediterranean diet interaction term in the analysis of the 33 candidate SNPs from the meta-analysis [56].

					Low AMD		High AMD	
SNP	CHR	Genes	MAF	*p*-GxD	Beta 1	SE1	Beta 2	SE2
rs12407512	1	intergenic	0.132	1.30 × 10^−2^	0.111	0.749	−2.613	0.801
rs3741475	12	*NOS1*	0.260	3.48 × 10^−2^	0.498	0.572	−1.360	0.669
rs2155281	11	*NCAM1*	0.352	3.81 × 10^−2^	0.699	0.510	−0.966	0.620
rs3793577	9	*ELAVL2*	0.499	1.50 × 10^−1^	0.063	0.478	−1.036	0.594
rs17432675	1	*LMOD1*	0.397	2.31 × 10^−1^	0.610	0.481	−0.341	0.633

AMD: Adherence to the MedDiet. CHR: Chromosome. Beta: Regression coefficient. *p*-GxD: *p*-value for the gene–MedDiet interaction term in the hierarchical linear regression model. MAF: Minor allele frequency in this population. Beta 1: Regression coefficients and SE for the SNP in the low level of adherence to the MedDiet. Beta 2: Regression coefficients and SE for the SNP in the high level of adherence to MedDiet. Low adherence (<9 points) and high (≥9 points).

## Data Availability

Neither the participants’ consent forms nor ethics approval included permission for open access. However, we follow a controlled data-sharing collaboration model, and data for collaborations will be available upon request, pending application and approval. Investigators who are interested in this study can contact the corresponding author.

## Supplementary Materials

The following supporting information can be downloaded at https://www.mdpi.com/article/10.3390/nu17233791/s1, Figure S1: Q-Q plot for the Genome-Wide Association Study (GWAS) of neuroticism adjusted for sex, age, diabetes, and BMI in this population; Figure S2: Gene expression heatmap in FUMA based on the data set GTExV8 showing the average expression for the DSCAM, FBLN2, and NDUFA10 genes in the GWAS for neuroticism; Figure S3: Zoom plot of the intergenic SNP rs1248033 in the GWAS of neuroticism.; Figure S4: Zoom plot of the intergenic SNP rs10753107 in the GWAS of neuroticism; Figure S5: Gene-based analysis in FUMA: (A) Manhattan plot; (B) enrichment of specific tissues; Figure S6: Frequency distribution of the GRS for the replication analysis; Table S1: SNPs for the replication of neuroticism, adjusted for sex, age, diabetes, and BMI; Figure S7: Manhattan plot of the SNP-based GWAS analyzing the interaction between the SNPs and adherence to Mediterranean diet for neuroticism; Table S2: Associated SNPs ranked by the *p*-value of the gene–Mediterranean diet interaction term in the analysis of the 33 candidate SNPs from the meta-analysis.
