# Supplementary material for: Linking Personality Traits to Mediterranean Diet Adherence and Exploring Gene–Diet Interactions in Neuroticism
Source: Nutrients. 2025 Dec 3;17(23):3791. doi: 10.3390/nu17233791 (PMC12693828; doi:10.3390/nu17233791)
Supplement: Supplementary file 1 [file nutrients-17-03791-s001.zip › nutrients-3993941-supplementary.pdf]

Article

Linking personality traits to the Mediterranean diet adherence and exploring gene-diet interactions in neuroticism

José V. Sorlí, Carolina Ortega-Azorín, Oscar Coltell, Rebeca Fernández-Carrión, Eva M. Asensio, Olga Portolés, Alejandro Perez-Fidalgo, Judith B. Ramirez-Sabio, Javier Guillem-Saiz, José A. Costa, Ignacio M. Gimenez-Alba, Rocío Barragán, Jose M. Ordovas, and Dolores Corella

SUPPLEMENTARY MATERIAL

Index

Figure S1. Q-Q plot for the Genome-Wide Association Study (GWAS) of Neuroticism adjusted for sex, age, diabetes and BMI in this population 2

Figure S2. Gene expression heatmap in FUMA based on the dataset GTExV8 showing the average expression for the DSCAM, FBLN2 and NDUFA10 genes in the GWAS for Neuroticism 2

Figure S3. Zoom plot of the intergenic SNP rs1248033 in the GWAS of Neuroticism. 3

Figure S4. Zoom plot of the intergenic SNP rs10753107 in the GWAS of Neuroticism 3

Figure S5. Gene-based analysis in FUMA: (A) Manhattan plot; (B) Enrichment of specific tissues 4

Figure S6. Frequency distribution of the GRS in this population 5

Table S1. SNPs fo r the replication of neuroticism, adjusted for sex, age, diabetes, and BMI 6

Figure S7. Manhattan plot of the SNP-based GWAS analyzing the interaction between the SNPs and adherence to Mediterranean Diet for Neuroticism 7

Table S2. Associated SNPs ranked by the p-value of the gene–Mediterranean diet interaction term in the analysis of the 33 candidate SNPs from the meta-analysis. 8

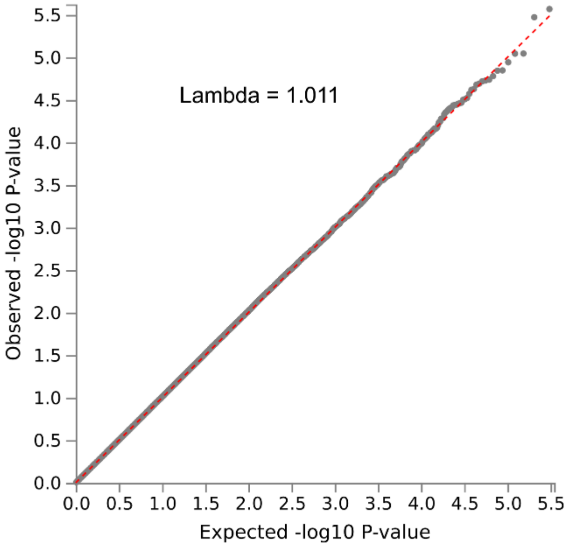

Figure S1. Q-Q plot for the Genome-Wide Association Study (GWAS) of Neuroticism adjusted for sex, age, diabetes and BMI in this population.

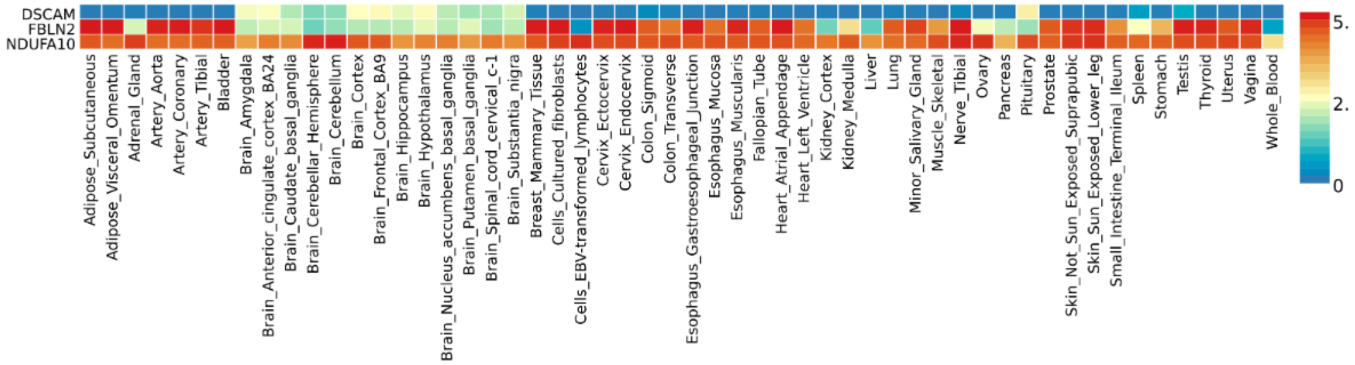

Figure S2. Gene expression heatmap in FUMA based on the dataset GTExV8 (54 tissue types), showing the average expression for the *DSCAM*, *FBLN2* and *NDUFA10* genes selected in the GWAS for Neuroticism.

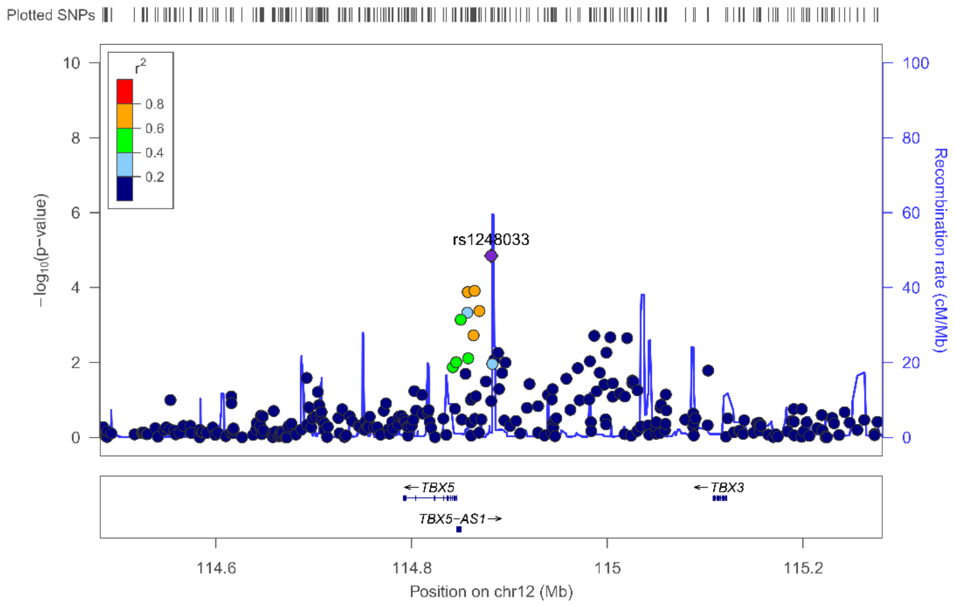

Figure S3. Zoom plot of the intergenic SNP, rs1248033, in the GWAS of Neuroticism in this population.

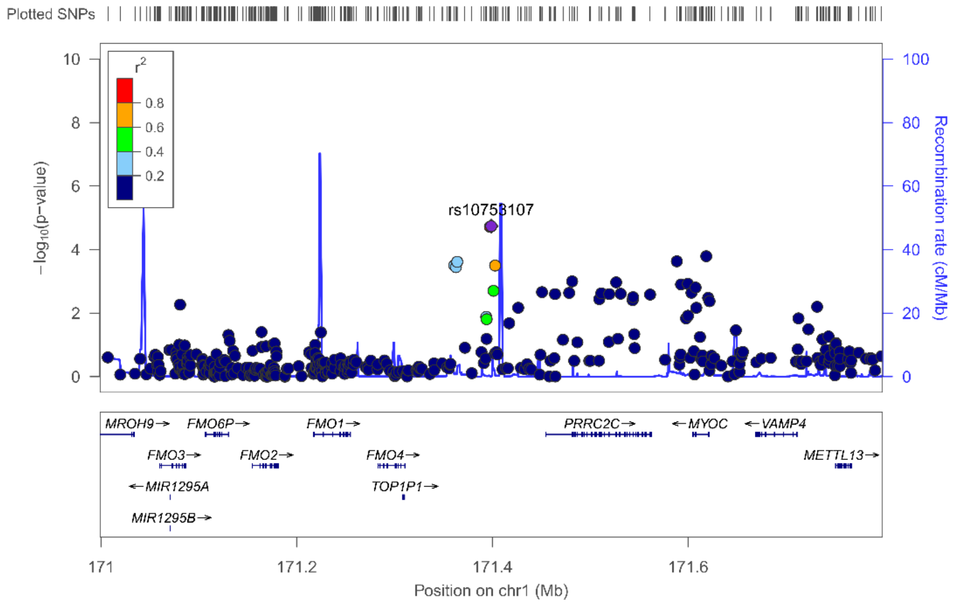

Figure S4. Zoom plot of the intergenic SNP, rs10753107, in the GWAS of Neuroticism in this population.

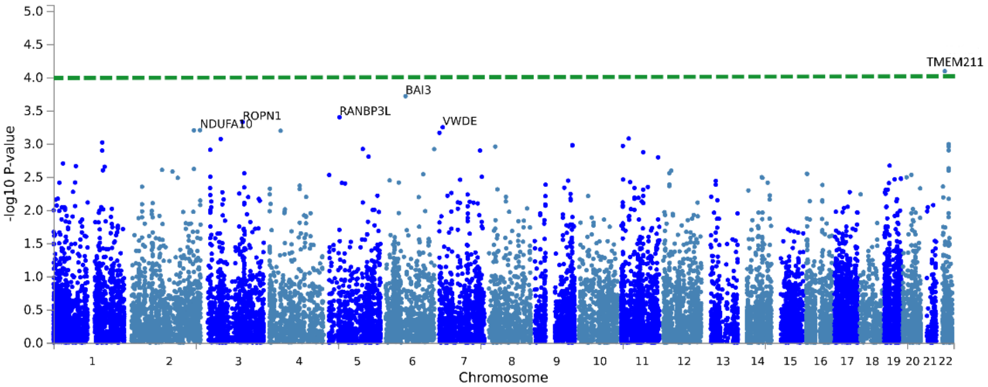

A

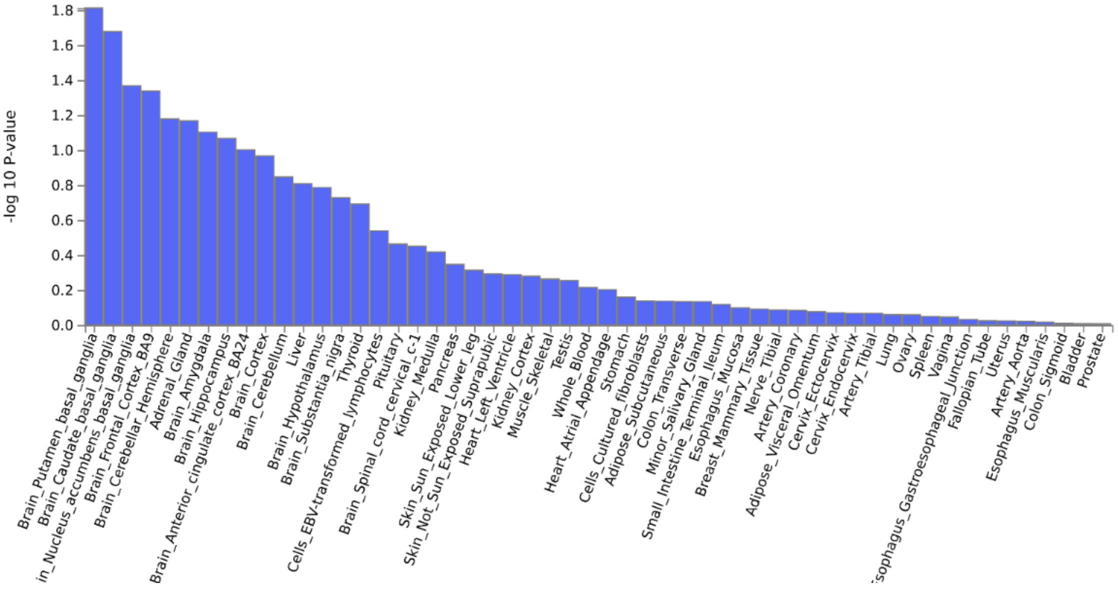

B

Figure S5. Gene-based analysis in FUMA: (**A**) Manhattan plot of the genes most significantly associated with Neuroticism in the GWAS model adjusted for sex, age, diabetes and BMI. The green line represents the threshold of suggestive significance ($p=1x10^{-4}$). The threshold of the strict Bonferroni corrected significance is 5.56 ($-\log_{10}(2.7x10^{-6})$); (**B**) Enrichment of specific tissues (MAGMA Tissue Expression Analysis based on the expression dataset of GTEx v8 53 tissue types) for the top-ranked genes in the GWAS for Neuroticism.

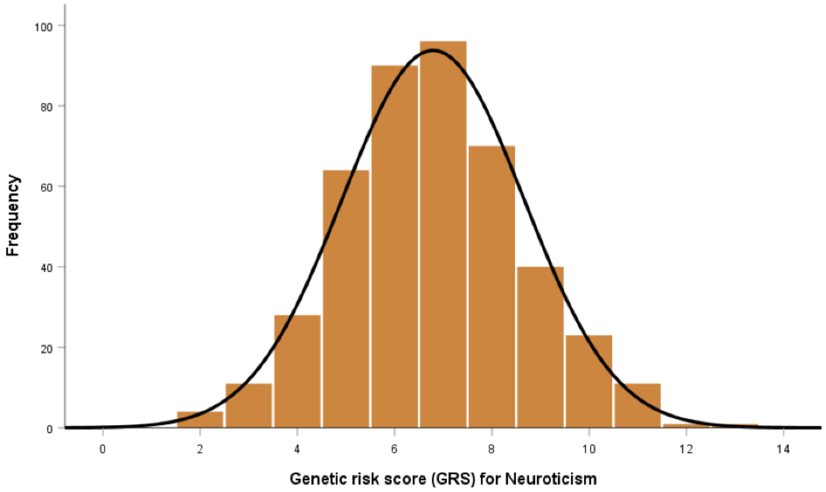

Figure S6. Frequency distribution of the scores for the genetic risk score for Neuroticism constructed in this population.

Table S1. SNPs for the replication of neuroticism, adjusted for sex, age, diabetes, and BMI [56]. SNPs adjusted for sex, age, diabetes and BMI, compared with the metanalysis of other populations.

SNP	CHR	BP	A1	Valencia MAF	Valencia Beta	P[1]	Genes	Meta EAF	Meta Beta	P[2]
rs12407512	1	217344002	C	0.132	-1.122	3.67x10^{-2}	intergenic	0.840	0.016	3.12x10^{-8}
rs2243873	6	31863433	C	0.352	-0.794	4.49x10^{-2}	*EHMT2*	0.575	0.012	3.29x10^{-8}
rs4585149	3	157493952	A	0.209	-0.864	4.83x10^{-2}	intergenic	0.177	-0.017	1.00x10^{-8}
rs1187257	18	35288227	G	0.243	0.805	6.03x10^{-2}	intergenic	0.715	-0.014	2.04x10^{-8}
rs7025144	9	120496387	A	0.255	0.792	6.29x10^{-2}	intergenic	0.272	0.018	5.20x10^{-13}
rs8929	11	45262126	G	0.420	0.666	7.86x10^{-2}	*SYT13*	0.526	0.012	4.13x10^{-8}
rs9671386	14	75151047	G	0.281	-0.636	1.06x10^{-1}	*AREL1*	0.744	0.020	1.04x10^{-16}
rs12938775	17	2574821	G	0.431	-0.537	1.54x10^{-1}	*PAFAH1B1*	0.505	-0.016	1.54x10^{-13}
rs10789942	11	113236199	A	0.415	-0.534	1.69x10^{-1}	*TTC12*	0.477	0.017	4.00x10^{-14}
rs3793577	9	23737627	A	0.499	-0.469	1.99x10^{-1}	*ELAVL2*	0.466	-0.015	4.43x10^{-11}
rs1542212	3	35683935	C	0.409	-0.453	2.31x10^{-1}	*ARPP21*	0.615	-0.018	3.97x10^{-16}
rs4362360	15	86940622	G	0.440	0.441	2.41x10^{-1}	*AGBL1*	0.532	0.013	5.45x10^{-9}
rs2073498	3	50369546	A	0.100	0.709	2.44x10^{-1}	*RASSF1*	0.111	0.021	3.76x10^{-10}
rs269101	1	112628860	A	0.367	-0.422	2.70x10^{-1}	intergenic	0.402	-0.012	4.15x10^{-8}
rs4396680	2	10178236	A	0.179	0.487	3.12x10^{-1}	intergenic	0.203	0.016	4.75x10^{-9}
rs1775703	1	116552570	A	0.374	0.355	3.58x10^{-1}	*SLC22A15*	0.314	-0.013	2.89x10^{-8}
rs3741475	12	117669914	A	0.260	-0.341	4.23x10^{-1}	*NOS1*	0.200	0.017	1.39x10^{-9}
rs2042395	16	87446053	G	0.261	-0.291	4.76x10^{-1}	*ZCCHC14*	0.755	-0.014	2.80x10^{-8}
rs2721939	8	116635942	G	0.379	0.269	4.98x10^{-1}	*TRPS1*	0.604	0.013	3.85x10^{-9}
rs4581549	12	24077866	A	0.125	-0.326	5.66x10^{-1}	*SOX5*	0.120	-0.020	4.41x10^{-9}
rs9867227	3	16866253	A	0.276	-0.221	6.02x10^{-1}	intergenic	0.283	0.018	8.93x10^{-14}
rs11238221	7	52736727	A	0.491	0.181	6.28x10^{-1}	intergenic	0.475	0.012	2.31x10^{-8}
rs8007859	14	69704553	C	0.398	-0.180	6.41x10^{-1}	*EXD2*	0.625	0.014	7.24x10^{-10}
rs2155281	11	112838338	A	0.352	-0.144	7.10x10^{-1}	*NCAM1*	0.396	0.018	1.63x10^{-15}
rs12705960	7	114015707	C	0.424	-0.133	7.20x10^{-1}	*FOXP2*	0.518	-0.012	2.05x10^{-8}
rs655836	3	136097576	G	0.388	0.131	7.32x10^{-1}	*STAG1*	0.577	0.017	1.47x10^{-14}
rs459358	1	44745624	A	0.277	-0.112	7.94x10^{-1}	*ERI3*	0.288	0.014	6.80x10^{-9}
rs2488401	1	197702401	A	0.218	-0.110	8.12x10^{-1}	*DENND1B*	0.209	-0.016	9.38x10^{-10}
rs12283653	11	46231998	G	0.431	0.072	8.49x10^{-1}	intergenic	0.608	-0.015	4.85x10^{-11}
rs6726405	2	124288380	A	0.410	-0.062	8.67x10^{-1}	intergenic	0.368	0.013	2.60x10^{-8}
rs17782313	18	57851097	G	0.218	0.072	8.68x10^{-1}	intergenic	0.765	0.015	9.40x10^{-10}
rs17432675	1	201887721	G	0.397	0.047	9.01x10^{-1}	*LMOD1*	0.674	0.013	4.00x10^{-8}
rs9267531	6	31636742	G	0.055	0.047	9.51x10^{-1}	*CSNK2B*	0.893	0.019	1.54x10^{-8}

CHR: Chromosome. BP: Base-pair. A1: Allele 1 (minor). EAF: Effect allele frequency. Beta: Regression coefficient. Valencia MAF: Minor allele frequency calculated in this population. Meta MAF: Minor allele frequency calculated in other (mixed) populations [56]. [1] P-value of the model adjusted for sex, age, diabetes and BMI. [2] P-value in the metanalysis of other populations [56].

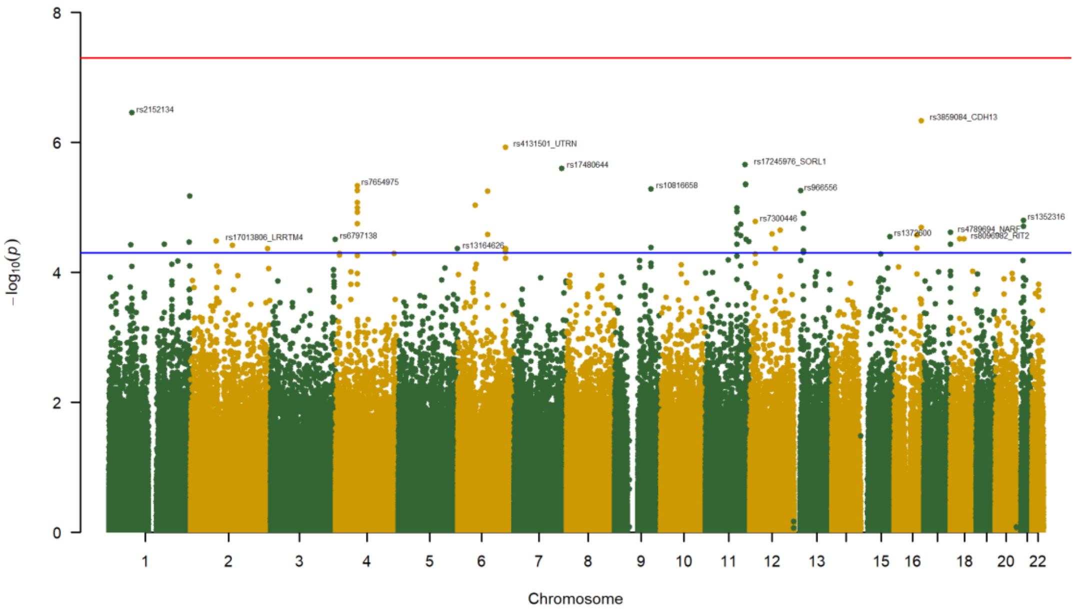

Figure S7. Manhattan plot of the SNP-based GWAS analyzing the interaction between the SNPs and adherence to Mediterranean Diet (as dichotomous variable of 2 categories: low and high) for Neuroticism. The red line represents the threshold 1 ($-\log_{10}(5\times10^{-8})$) for the GWAS statistical significance. The blue line represents the threshold 2 ($-\log_{10}(5\times10^{-5})$) for the suggestive significance.

Table S2. Associated SNPs ranked by the p-value of the gene–Mediterranean diet interaction term in the analysis of the 33 candidate SNPs from the meta-analysis [56].

SNP	CHR	Genes	MAF	P_GxD	Bp
rs12407512	1	intergenic	0.132	$1.30x10^{-2}$	217344002
rs3741475	12	*NOS1*	0.260	$3.48x10^{-2}$	117669914
rs2155281	11	*NCAM1*	0.352	$3.81x10^{-2}$	112838338
rs3793577	9	*ELAVL2*	0.499	$1.50x10^{-1}$	23737627
rs17432675	1	*LMOD1*	0.397	$2.31x10^{-1}$	201887721
rs9267531	6	*CSNK2B*	0.055	$2.84x10^{-1}$	31636742
rs6726405	2	intergenic	0.410	$2.94x10^{-1}$	124288380
rs9671386	14	*AREL1*	0.281	$3.96x10^{-1}$	75151047
rs11238221	7	intergenic	0.491	$4.20x10^{-1}$	52736727
rs8929	11	*SYT13*	0.420	$4.36x10^{-1}$	45262126
rs2488401	1	*DENND1B*	0.218	$4.51x10^{-1}$	197702401
rs1542212	3	*ARPP21*	0.409	$4.67x10^{-1}$	35683935
rs2243873	6	*EHMT2*	0.352	$4.78x10^{-1}$	31863433
rs2073498	3	*RASSF1*	0.100	$5.10x10^{-1}$	50369546
rs12938775	17	*PAFAH1B1*	0.431	$5.20x10^{-1}$	2574821
rs269101	1	intergenic	0.367	$5.88x10^{-1}$	112628860
rs1187257	18	intergenic	0.243	$5.92x10^{-1}$	35288227
rs9867227	3	intergenic	0.276	$6.16x10^{-1}$	16866253
rs12283653	11	intergenic	0.431	$6.16x10^{-1}$	46231998
rs8007859	14	*EXD2*	0.398	$6.21x10^{-1}$	69704553
rs459358	1	*ERI3*	0.277	$6.31x10^{-1}$	44745624
rs17782313	18	intergenic	0.218	$6.72x10^{-1}$	57851097
rs4396680	2	intergenic	0.179	$6.90x10^{-1}$	10178236
rs1775703	1	*SLC22A15*	0.374	$7.39x10^{-1}$	116552570
rs7025144	9	intergenic	0.255	$7.60x10^{-1}$	120496387
rs655836	3	*STAG1*	0.388	$7.81x10^{-1}$	136097576
rs4362360	15	*AGBL1*	0.440	$8.25x10^{-1}$	86940622
rs4585149	3	intergenic	0.209	$8.48x10^{-1}$	157493952
rs2042395	16	*ZCCHC14*	0.261	$8.54x10^{-1}$	87446053
rs10789942	11	*TTC12*	0.415	$8.59x10^{-1}$	113236199
rs2721939	8	*TRPS1*	0.379	$8.75x10^{-1}$	116635942
rs4581549	12	*SOX5*	0.125	$8.87x10^{-1}$	24077866
rs12705960	7	*FOXP2*	0.424	$9.58x10^{-1}$	114015707

CHR: Chromosome. P-GxD: P-value for the gene-MedDiet interaction term in the hierarchical linear regression model. MAF: Minor allele frequency calculated in this population. Bp: Base-pairs. [1] P-value for the interaction term.
